# Human Fetal Astrocytes Infected with Zika Virus Exhibit Delayed Apoptosis and Resistance to Interferon: Implications for Persistence

**DOI:** 10.3390/v10110646

**Published:** 2018-11-17

**Authors:** Daniel Limonta, Juan Jovel, Anil Kumar, Adriana M. Airo, Shangmei Hou, Leina Saito, William Branton, Gane Ka-Shu Wong, Andrew Mason, Christopher Power, Tom C. Hobman

**Affiliations:** 1 Department of Cell Biology, University of Alberta, Edmonton, Alberta, T6G 2H7, Canada; dlimonta@ualberta.ca (D.L.); anilkuma@ualberta.ca (A.K.); shangmei@ualberta.ca (S.H.); 2 Department of Medicine, University of Alberta, Edmonton, Alberta, T6G 2E1 Canada; jovel@ualberta.ca (J.J.); leina@ualberta.ca (L.S.); wbranton@ualberta.ca (W.B.); gane@ualberta.ca (G.K.-S.W.); am16@ualberta.ca (A.M.); chris.power@ualberta.ca (C.P.); 3 Department of Medical Microbiology Immunology, University of Alberta, Edmonton, Alberta, T6G 2E1 Canada; airo@ualberta.ca; 4 Department of Biological Sciences, University of Alberta, Edmonton, Alberta, T6G 2E9, Canada; 5 Beijing Genomics Institute, Beishan Industrial Zone, Yantian District, Shenzhen, 518083, China; 6 Women Children’s Health Research Institute, University of Alberta, Edmonton, Alberta, T6G 1C9, Canada; 7 Li Ka Shing Institute of Virology, University of Alberta, Edmonton, Alberta, T6G 2E1, Canada

**Keywords:** Zika virus, persistence, astrocytes, interferon, apoptosis

## Abstract

Zika virus (ZIKV) infection and persistence during pregnancy can lead to microcephaly and other fetal neurological disorders collectively known as Congenital Zika Syndrome. The immunological and virological events that contribute to the establishment of persistent ZIKV infection in humans are unclear though. Here we show that human fetal astrocytes (HFAs), the most abundant cell type in the central nervous system, become persistently infected with ZIKV resulting in continuous viral shedding for at least one month; a process that is facilitated by TIM/TAM receptors. HFAs are relatively resistant to ZIKV-induced apoptosis, a factor that may be important for chronic infection of these cells. Once infection was established, interferon treatment did not reduce virus replication. Moreover, the fact that the innate immune system was highly activated in persistently infected HFAs indicates that the virus can thrive in the presence of a sustained antiviral response. RNAseq analyses of persistently infected cells revealed that ZIKV alters host gene expression in a manner that could affect developmental processes. Conversely, data from sequencing of ZIKV genomes in persistently infected HFAs suggest that adaptive mutations were not required for establishing chronic infection. Based on these results, we postulate that HFAs are reservoirs for ZIKV in the fetal brain and that moderate apoptosis combined with inefficient antiviral response from these cells may contribute to the establishment of chronic brain infection associated with the ZIKV neurodevelopmental abnormalities.

## 1. Introduction

Zika virus (ZIKV) is an arthropod-borne virus of the genus Flavivirus, family *Flaviviridae*. It was first isolated approximately 70 years ago in the Zika forest of Uganda [1]. The virus spread throughout Africa and South Asia but other than an undifferentiated febrile syndrome with rash and arthralgia, ZIKV infections were of little consequence. However, the recent ZIKV pandemic in the Western Hemisphere was associated with a dramatic increase in microcephaly and other neurological deficits in the fetuses [2,3]. Prolonged ZIKV infection of fetal cells and tissues that serve as reservoirs for the virus are needed to establish persistence during pregnancy. A number of animal and in vitro models describing the tropism and pathogenesis of the ZIKV infection have recently been published [4,5,6] but the immune and viral mechanisms involved in chronic infection of human fetal brain cells are not completely understood. ZIKV has been shown to chronically infect neuroprogenitor cells derived from induced pluripotent human stem cells [7] in which it induces cell death and dysregulation of the cell cycle [8,9].

Astrocytes are the first brain cell type to be targeted by ZIKV after peripheral inoculation of newborn mice [10] with interferon (IFN) systems playing a major role in restricting viral replication [10,11]. Reactive astrocytes (astrogliosis) have been reported in brain tissue of an adult mice model of ZIKV [12] and in human fetuses with Congenital Zika Syndrome [13]. AXL, a TAM family receptor that is highly expressed in stem cell-derived glia including astrocytes, was proposed as a candidate for mediating ZIKV entry into fetal brain cells [14]. Subsequently, it was shown to function as a ZIKV entry receptor during acute infection of human astrocytes derived from stem cells in vitro [15] and primary human astrocytes [16]. More recently, it has been proposed that AXL promotes ZIKV infection of human astrocytes by antagonizing the IFN response [17]. Despite these important findings, the impact of persistent ZIKV infection on *bona fide* human fetal astrocytes (HFAs) has not been thoroughly investigated.

Here, we examined the potential importance of resistance to apoptosis and the IFN response in chronic infection of HFAs. Primary HFAs were highly permissive to ZIKV, a process that was dependent upon the TIM/TAM receptor member AXL. Compared to continuous human cell lines, viral infection of HFAs resulted in relatively low-levels of apoptosis. Addition of IFN did not block chronic viral infection and infected HFAs continued to shed virus for at least one month despite the robust antiviral response. To gain further understanding of how prolonged ZIKV infection affects gene expression in HFAs, we performed transcriptomic analyses of persistently-infected HFAs and identified multiple cellular pathways that are affected by the virus. This is the first demonstration that ZIKV can persist in *bona fide* HFAs *ex vivo* for prolonged periods of time. Together, our data provide novel insights into how ZIKV establishes persistent infection in the fetal brain and how this may affect cellular processes leading to neuropathogenesis.

## 2. Materials and Methods

### 2.1. Ethics Statement

Human fetal brain tissues were obtained from 15 to 19-week aborted fetuses with written consent from the donor under the protocol 1420 by the University of Alberta Human Research Ethics Board (identification code Pro00027660, approved on 13 May 2012).

### 2.2. Virus Strains and Cell Lines

A low passage Asian lineage ZIKV strain (PLCal ZV) isolated from a Canadian traveler in 2013 [18] and the prototype Asian ZIKV strain isolated in Puerto Rico (PRVABC-59) in 2015 [19] were obtained from the Public Health Agency of Canada. The African virus strain (MR766) was generated from an infectious clone of the 1947 Uganda ZIKV kindly donated by Dr. Matthew J. Evans at the Icahn School of Medicine at Mount Sinai, New York [20]. Viruses were propagated in *Aedes albopictus* C6/36 cells grown in Minimum Essential Medium (MEM) supplemented with 10% fetal bovine serum (Gibco, Waltham, MA, USA), l-glutamine, Penicillin-Streptomycin and MEM non-essential amino acids at 32 °C.

Viral stocks for all the experiments were prepared after inoculating C6/36 cells with the multiplicity of infection (MOI) of 0.2 and harvesting supernatants at 48 and 96 h post-infection. Virus-containing media were clarified by centrifugation at 3200× *g* for 10 min as previously described [21]. HFAs were isolated from brain tissue obtained from 15–19 week aborted fetuses as previously described [22]. HFAs were grown in MEM (1 g/L Glucose, 15mM HEPES, Gibco) supplemented with 10% fetal bovine serum (Gibco), l-glutamine, MEM non-essential amino acids, sodium pyruvate, and 1 g/mL glucose. For all experiments, HFAs cultures between 5–7 passages were employed. A549 (human lung carcinoma), Vero (African green monkey kidney) and U373 (human astrocytoma) cells from the American Type Culture Collection (Manassas, VA, USA) were maintained in Dulbecco’s Modified Eagle Medium (DMEM) supplemented with 10% fetal bovine serum, 15 mM HEPES (Gibco), l-glutamine and Penicillin-Streptomycin.

### 2.3. Virus Infection

HFAs or A549 cells were seeded in 6-well plates at 4–5 × 10^5^ cells per well (Greiner, Kremsmünster, Austria) or 96-wells plates (CELLSTAR, Radnor, USA) at 1 × 10^4^ cells per well. Cells were rinsed once with PBS and ZIKV at an MOI of 0.3–10 was added to the cells. Cells then were incubated for 2 h at 37 °C using fresh media supplemented with 3% fetal bovine serum (Gibco). Next, the inoculum was removed and the cells were washed twice with PBS. Complete culture medium was added to each well, and cells were incubated at 37 °C and 5% CO_2_. Mock cells were incubated with the culture supernatant from uninfected C6/36 cells. For viral kinetics, cells were incubated in 24-well plates (Greiner) at 5 × 10^4^ cells per well.

### 2.4. ZIKV Titration

ZIKV was serially diluted (10-fold dilutions) and infected monolayers of Vero cells at 37 °C for 2 h. The monolayers were overlaid with a mixture of MEM (Gibco) and 1.5% carboxymethylcellulose (Sigma-Aldrich, St. Louis, MO, USA) following the infection. The cells were maintained at 37 °C for 4 days for plaque development. Before plaque counting, cells were fixed with 10% formaldehyde and stained with 1% crystal violet in 20% ethanol.

### 2.5. Quantitative Reverse-Transcription PCR Assay

For cellular mRNA and ZIKV RNA analysis, total RNA was extracted using RNeasy mini kit (QIAGEN, Valencia, CA, USA) following the manufacturer’s protocol. RNA extraction from cells of the viral persistence assay was done with NucleoSpin RNA kit (Macherey-Nagel, Düren, Germany). RNA samples were treated with RNase-free DNase (QIAGEN or Macherey-Nagel). Total RNA was reverse transcribed using 0.5–1 µg of total RNA and ImProm-II Reverse Transcriptase (Promega, Madison, WI, USA) according to the manufacturer’s protocol. qRT-PCR was performed with PerfeCTa SYBR Green SuperMix (Quanta BioSciences, Beverly, MA, USA) in an Mx3000P qPCR Systems (Agilent Technologies, Santa Clara, CA, USA). The cycling conditions were 45 cycles of 94 °C for 30 s, 55 °C for 60 s, and 68 °C for 20 s. Gene expression (fold change) was calculated using the 2^(−ΔΔCT)^ method using human β-actin mRNA transcript as the internal control. Primer sequences used for RT-PCR are shown in Table S1.

### 2.6. Enzyme-Linked Immunosorbent Assay

To measure secreted NS1, supernatants from mock or ZIKV-infected HFAs were collected at indicated time points (1–4 days post-infection). NS1 levels were measured using a horse-radish peroxidase-conjugated anti-NS1 mouse monoclonal antibody developed in this laboratory [23]. NS1 levels were determined using a standard curve generated against known quantities of ZIKV NS1 purchased from The Native Antigen Company Ltd (Oxford, UK).

### 2.7. Cell Viability Assay

CellTiter-Glo Luminescent Cell Viability Assay (Promega) was used to measure ATP levels in HFAs and A549 cells. Cells were assayed after mixing 100 µL of complete media with 100 µL of reconstituted CellTiter-Glo Reagent (buffer plus substrate) following the manufacturer’s instructions. Plates were shaken and luminescence was recorded, 10 min after adding the reagent, with a GloMax Explorer Model GM3510 (Promega).

### 2.8. Immunofluorescence Staining and Imaging Analysis

Cells on coverslips or 96-well plates (CELLSTAR) were fixed for 15 min at room temperature with 4% paraformaldehyde in PBS. Cells were then washed three times in PBS and then permeabilized/blocked with 0.2% Triton-X100 and 3% BSA in PBS for 1 h at room temperature followed by washing with PBS containing 0.3% BSA. Incubations with primary antibodies diluted 1:500 (mouse anti-Flavivirus Group Antigen 4G2, Millipore, Burlington, MA, USA), and 1:500 (rabbit anti-glial fibrillary acidic protein (GFAP), Dako, Santa Clara, CA, USA) in blocking buffer (3% BSA and PBS) were carried out at room temperature for 1.5 h followed by three washes in 0.02% Triton-X100 with 0.3% BSA and PBS. Samples were then incubated with secondary antibodies (1:1000) in blocking buffer containing 1 µg/mL of DAPI for 1 h at room temperature followed by three washes in 0.3% BSA in PBS. Secondary antibodies (Invitrogen, Waltham, MA, USA) were Alexa Fluor 488 donkey anti-mouse, and Alexa Fluor 546 donkey anti-rabbit. Confocal images of HFAs on coverslips were acquired using an Olympus 1 × 81 spinning disk confocal microscope (Tokyo, Japan) and images were analyzed using Volocity 6.2.1 software (PerkinElmer, Waltham, MA, USA). Images of HFAs or A549 cells on 96-well plates were acquired using the Operetta High Content Imaging System (Perkin Elmer, Waltham, MA, USA) with 20× objective lens, and analyzed using Harmony 3.5 software (Waltham, MA, USA). Total and antigen-positive cells were counted in 15 image fields per well, and the percentage of infected cells was obtained.

### 2.9. Flow Cytometry

Mock- or infected-HFAs and A549 cells were harvested at indicated time points by trypsin detachment, counted with a Moxi Z mini automated cell counter (Orflo, Ketchum, ID, USA), fixed (4% paraformaldehyde in PBS), permeabilized (0.1% saponin in PBS), and stained with a rabbit antibody that recognizes cleaved caspase-3 (Cell Signaling, Danvers, MA, USA) and mouse monoclonal antibody anti-Flavivirus Group Antigen 4G2 (Millipore) that recognizes flavivirus envelope proteins followed by chicken anti-rabbit Alexa Fluor 594 and goat anti-mouse Alexa Flour 405 labeled secondary antibody (Invitrogen). Staurosporine (Cell Signaling) treatment was used as a positive control to induce apoptosis in A549 cells. Samples were subjected to analyses by flow cytometry (BD LSRFortessa X-20, BD Biosciences, San Jose, CA, USA) using BD FACSDiva V8.0.1 software. For detection of GFAP, cells were detached from the plates 2, 15, 21 and 28 days after seeding and then fixed and permeabilized as described above. Rabbit anti-GFAP (Dako) was the primary antibody and this was detected using chicken anti-rabbit Alexa Fluor 594 labeled antibody (Invitrogen). For detection of AXL, cells were detached using Versene (Sigma-Aldrich), followed by incubating on ice with goat anti-AXL (AF154, RD Systems, Minneapolis, MN, USA) and then chicken anti-goat Alexa Fluor 594 labeled antibody (Invitrogen). Samples were subjected to flow cytometry as described above. 

### 2.10. Virus Entry Inhibition Assays

ZIKV inoculum was incubated with medium containing duramycin (0.3–1.5 μM, Sigma-Aldrich) for 1 h, and then added to A549 cells and HFAs (MOI of 0.5) in 24-well plates (Greiner) for 2 h at 37 °C, 5% CO_2_. Next, equal amounts of media were added to each well and then cells were incubated for 24 h before levels of ZIKV genomic RNA were determined by qRT-PCR. For indirect immunofluorescence, infected A549 cells and HFAs in 96-well plates (CELLSTAR) were fixed (4% paraformaldehyde in PBS) after 24 h and then incubated with mouse anti-Flavivirus Group Antigen 4G2 antibody (Millipore) and Alexa Fluor 488 donkey anti-mouse antibody (Invitrogen) prior to image acquisition with an Operetta High Content Imaging System (Perkin Elmer) with 20× objective lens. Images were analyzed using Harmony 3.5 software. Total and ZIKV envelope protein-positive cells (in 15 image fields per well) were counted and the percentage of infected cells was obtained. To inhibit the kinase activity of AXL during ZIKV infection, A549 cells and HFAs were incubated with R428 (1.0–4.0 μM, Selleckchem, Houston, TX, USA) for 1 h at 37 °C, 5% CO_2_, then replaced with R428-containing medium with ZIKV (MOI of 0.5). After 2 h at 37 °C, medium was added to each well. Cells were incubated for 24 h, fixed, and processed for qRT-PCR or indirect immunofluorescence as above.

HFAs or A549 cells were treated with a goat anti-AXL-blocking antibody (AF154, RD Systems) at 100 μg/mL or non-immune goat IgG for 1 h prior to infection with ZIKV. After the antibody-blocking step, cells were washed once with PBS, infected with ZIKV (MOI = 10) and then cultured for 24 h before isolation of total RNA for qRT-PCR. Where indicated, persistently infected HFAs (2-weeks post-infection) were cultured with goat anti-AXL-blocking antibody (4 μg/mL) or non-immune goat IgG with media and antibody replenishment 2 days later until samples were collected for analyses the next day.

### 2.11. Viral Persistence Assay

For the viral persistence assay, HFAs were incubated in 6-well plates at 3 × 10^5^ cells per well, 12-well plates with coverslips at 1 × 10^5^ cells per well, and 24-well plates (Greiner) at 5 × 10^4^ cells per well. Cells were infected with ZIKV PLCal, MR766 or PRVABC59 at MOI of 0.3 or 3. Media were replaced every three days and samples (supernatants, cell lysates or cells on cover slips) were collected 24 h after media replacement.

### 2.12. Statistical Analyses

A paired Student’s *t*-test was used for pair-wise statistical comparisons. The means ± standard error of the mean are shown in all bar and line graphs. All statistical analyses were performed using GraphPad Prism software.

### 2.13. RNAseq Libraries

RNAseq libraries were constructed using a TruSeq RNA Sample Prep V2 Kit (Illumina, San Diego, CA, USA) as per manufacturer instructions. In brief, 1 μg of total RNA was diluted in 50 μL of nuclease-free H_2_O and mixed with one volume of messenger RNA purification beads containing oligos dT conjugated to paramagnetic beads and the suspension was heated to 65 °C for 5 min, cooled down to 4 °C and then incubated at room temperature for 5 min. The suspension was then incubated on a magnetic stand for 5 min at room temperature, the supernatant removed, and the beads washed with 200 μL of beads washing solution. Finally, the RNA was eluted from the beads in 50 μL of Elution Buffer at 85 °C, for 2 min and then cooled to 25 °C. RNA was re-bound to magnetic beads to increase specificity by adding 50 μL of Bead Binding Buffer after which beads were washed as described above. For the cDNA synthesis step, bead-bound RNA was supplemented with 19.5 μL Elute, Prime Fragment mix containing random hexamers. The solution was heated at 94 °C for 8 min and then cooled to 4 °C to allow for annealing of random hexamers. cDNA synthesis (8 μL volume) was performed in First Strand Master Mix containing SuperScript II reverse transcriptase at a 1:9 ratio using the following thermocycling program: 25 °C for 10 min, 42 °C for 50 min, 70 °C for 15 min, hold at 4 °C. Second strand cDNA synthesis reactions were performed in 25 μL of Second Strand Master Mix at 16 °C for 1 h. cDNA reactions were supplemented with 90 μL of AMPure XP beads, incubated for 15 min at room temperature, and then incubated for 5 min on a magnetic stand. Beads were washed twice with 200 μL of 80% ethanol, air-dried, and finally the cDNA was eluted in 50 μL Resuspension Buffer. End repair was conducted in 40 μL of End Repair Mix for 30 min at 30 °C. End-repaired cDNA was supplemented with 160 μL of AMPure XP beads, which were washed and end-repaired cDNA eluted in 15 μL as described above. End-repaired cDNA was 3′ adenylated in 12.5 μL of A-tailing mix with the following thermo cycler program: 37 °C for 30 min, 70 °C for 5 min, 4 °C hold. Adapters were ligated by adding 2.5 μL of Ligation Mix, incubated at 30 °C for 10 min; reaction was stopped with 5 μL of Stop Ligation Buffer. Reaction was cleaned up twice, in the first round with 42 μL of AMPure XP Beads and eluted in 50 μL of Resuspension Buffer, and in the second round with 50 μL AMPure XP Beads and eluted in 20 μL, all as described above. cDNA was enriched by adding 25 μL of PCR Master Mix using the following PCR program: 98 °C for 30 s; (15x) 98 °C for 10 s, 60 °C for 30 s, 72 °C for 30 s; 72 °C for 5 min; 4 °C hold. PCR reaction was cleaned up with 50 μL of AMPure XP Beads and recovered in 30 μL of Resuspension Buffer, as described above. Libraries were sequenced in a NextSeq instrument, using a 75 cycle paired end V3 sequencing kit (Illumina), at the depths indicated in Table S2.

### 2.14. Bioinformatics

Libraries were demultiplexed using the appropriate workflow in the NextSeq instrument (Illumina). After quality control trimming, libraries were pseudo-aligned to the GRCh38.81 version of the human genome with Kallisto [24]. Differential expression analysis was conducted with the programming language R, using the Sleuth package [25]. Plots were generated with R scripts. For analysis of HFAs genes that were deregulated, we conducted gene ontology analysis with the Cytoscape plug-in clueGO [26]. For determination of the mutations rate in the ZIKV genome, including single nucleotide polymorphisms (SNPs) and length polymorphisms (LPs), the software Vphaser-2 was used [27]. A Kolmogorov–Smirnov test was applied on the frequency of each type of mutations consolidated in segments of 500 nt.

### 2.15. Accession Number

The accession number for the raw data for the transcriptional response of HFAs to chronic Zika virus infection reported in this work have been deposited to the SRA of NCBI to be publicly available under the accession number: PRJNA356760.

## 3. Results

### 3.1. ZIKV Infects TIM/TAM-Expressing Cells

Before proceeding to infection studies, we first confirmed the presence of *bona fide* HFAs in our primary cell cultures. The purity of HFAs cultures was assessed by staining for GFAP [28]. By flow cytometry analysis, the vast majority (95%) of the cells in the HFA cultures were positive for GFAP expression (Figure S1A).

**Figure 1:**
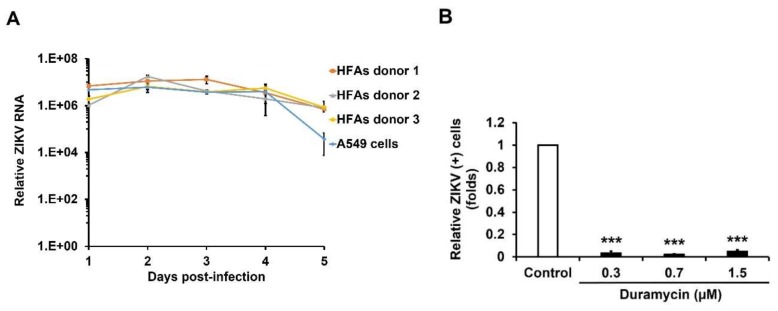

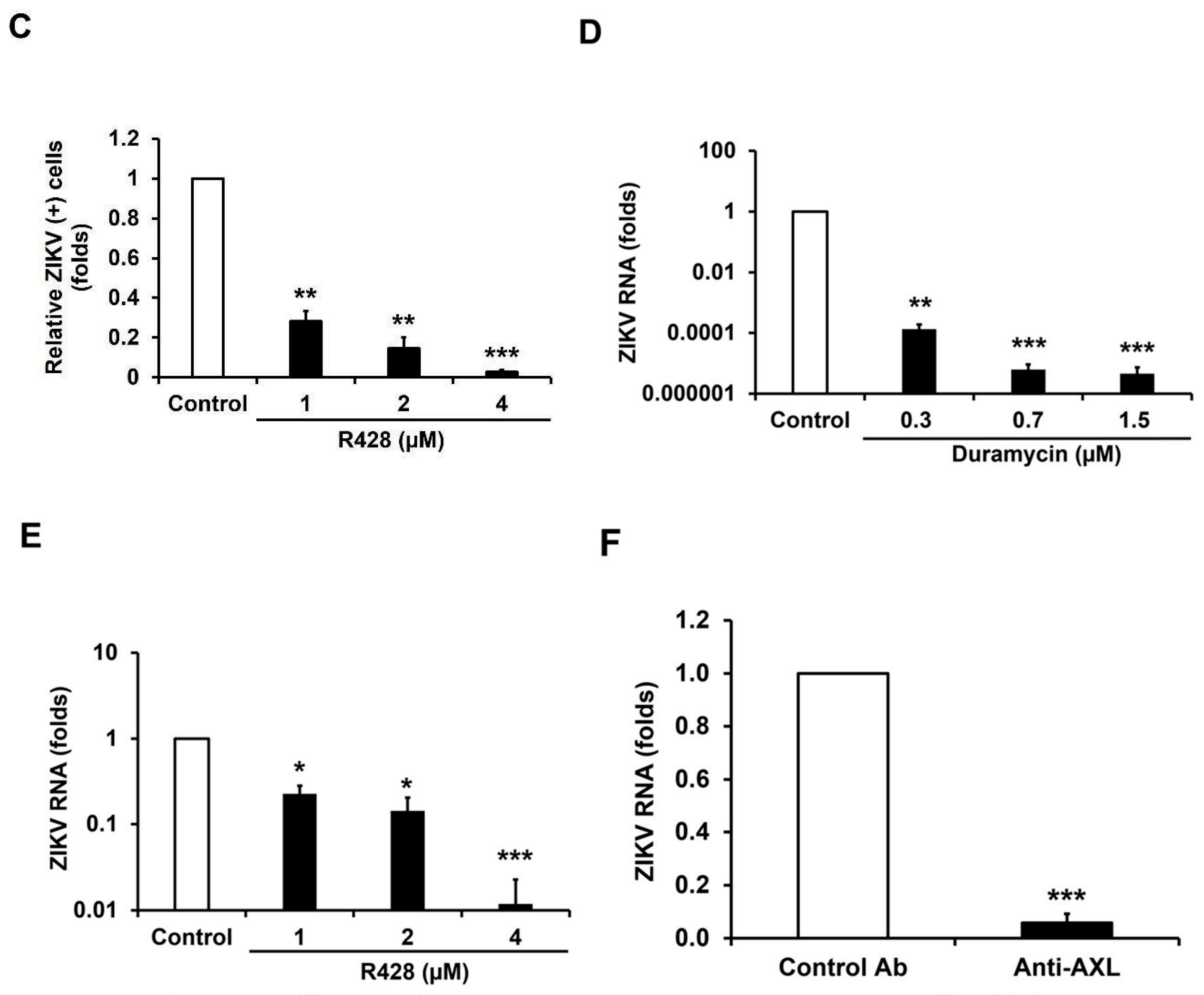
HFAs are highly permissive for ZIKV infection. (**A**) HFAs and A549 cells were infected with PLCal ZIKV (MOI = 3) for 1–5 days and virus replication was quantitated by measuring genomic RNA in cells using qRT-PCR. (**B**–**E**) Cells were infected with ZIKV that had been pre-incubated with duramycin for 1 h or first treated with R428 for 1 h followed by infection with ZIKV (MOI = 0.5). One day later, cells were processed for indirect immunofluorescence using antibodies against flavivirus envelope protein or total RNA was harvested for qRT-PCR. (**B**,**C**) Data from automated quantitation of ZIKV-positive cells. (**D**,**E**) Quantification of ZIKV replication by qRT-PCR after drug treatment. (**F**) HFAs were pre-incubated with anti-AXL blocking antibody for 1 h followed by ZIKV infection (MOI = 10). After 24 h, cells were collected and viral RNA was quantified by qRT-PCR. The means of three independent experiments are shown. Error bars represent standard error of the mean. * *p* 0.05, ** *p* 0.01, *** *p* 0.001 (Student’s *t*-test).

Next, HFAs were infected with ZIKV (MOI = 3) and replication was assessed by measuring viral genomic RNA 1–5 days post-infection. The levels of viral RNA in HFAs were comparable to those seen in the highly permissive human cell line A549 (Figure 1A). Production of ZIKV RNA in HFAs were highest at 2–3 days post-infection; however, secretion of the viral protein NS1 continued to increase for at least 4 days (Figure S1B).

Next, we addressed whether TIM/TAM family receptors [29] which bind phosphatidylserine and phosphatidyl ethanolamine, were important for infection of HFAs. Whereas the majority of A549 cells were positive for expression of AXL, depending upon the donor, only 23–46% of HFAs exhibited detectable levels of this protein (Figure S1C). Moreover, cell surface expression of AXL was lower in HFAs compared to A549 cells (Figure S1D). HFAs were treated with R428 (specific inhibitor for AXL) followed by infection with ZIKV or cells were infected with ZIKV that had been pre-incubated with duramycin (a cyclic peptide that binds phosphatidyl ethanolamine on the virion surface). Automated high-content immunofluorescence analyses revealed that both duramycin and R428 significantly reduced ZIKV infection of HFAs in a dose-dependent manner (Figure 1B,C and Figure S2A,B). Similarly, levels of viral RNA in drug-treated HFAs were also dramatically reduced (Figure 1D,E). HFAs viability was not significantly affected by concentrations of R428 and duramycin that showed antiviral activity (Figure S3A,B). Finally, ZIKV replication was dramatically reduced in HFAs that were pre-treated with an antibody to AXL further confirming the importance of this protein for viral entry and/or replication in *bona fide* HFAs (Figure 1F). Similar results were observed in A549 cells treated with AXL-blocking or activity reducing reagents (Figure S3C-I). 

### 3.2. Kinetics of ZIKV Persistent Infection in HFAs

**Figure 2:**
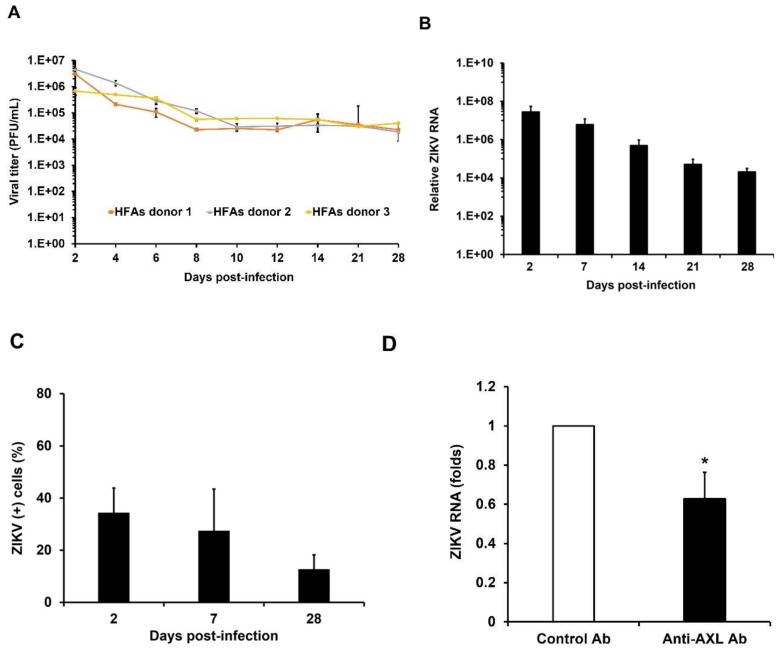
ZIKV can persistently infect primary HFAs. (**A**–**C**) HFAs were infected with ZIKV (MOI = 3) and media were collected at the indicated intervals for up to 28 days. Viral titers were determined by plaque assay. The percentages of infected cells at indicated time periods were determined by indirect immunofluorescence using antibodies against flavivirus envelope protein. (**A**) ZIKV titers in persistently infected HFAs. The average titers obtained from two experiments using three independent donors are shown. (**B**) Measurement of viral replication by qRT-PCR. The average values obtained from two experiments using two independent donors are shown. (**C**) Percentage of infected HFAs over time is shown. (**D**) Importance of viral spread in maintaining chronic infection. HFAs infected with ZIKV for 14 days were treated with anti-AXL (4 µg/mL) for 4 days and viral replication was determined by qRT-PCR. The average values obtained from three experiments using two independent donors are shown. Error bars represent standard error of the mean. * *p* 0.05 (Student’s *t*-test).

It is likely that ZIKV-induced neurological defects in the fetus are related to the ability of the virus to persist in the fetal brain for prolonged periods. Accordingly, we investigated whether primary HFAs can be chronically infected with a contemporary ZIKV strain of the Asian lineage (PLCal ZV) [18]. Following the acute infection period, we observed that HFAs continuously produced moderate levels (10,000 pfu/mL) of ZIKV for at least one month (Figure 2A). The viral titer data were consistent with qRT-PCR-based viral replication analyses (Figure 2B). Microscopic analyses of the persistently infected HFAs revealed that ~10% of the cells were positive for viral antigen 28 days post-infection (Figure 2C, Figure S4A,B). The astrocytic phenotype of cultures was verified by flow cytometry in which over 90% of the cells were GFAP-positive for one month (Figure S4C).

We also assessed whether HFAs could be persistently infected with the African (MR766) and the Centers for Disease Control reference (PRVABC59) strains of ZIKV. Similar to HFAs infected with the PLCal ZV strain, astrocytes infected with MR766 and PRVABC59 produced sustained viral titers in excess of 10^5^ pfu/mL over the 4-week infection period (Figure S4D,E). Even when infected with a much lower MOI (0.3) of the microcephalic ZIKV strain PRVABC59, HFAs continually produced relatively high viral titers for at least one month (Figure S4F). These data indicate that HFAs can be chronically infected with old and new world strains of ZIKV.

### 3.3. AXL Mediates Viral Spread During ZIKV Persistence 

Because only a fraction of the cells in infected HFAs cultures exhibited detectable ZIKV antigen, we asked whether continuous viral spread was required to maintain persistence and if so, whether AXL receptors were involved in this process. Chronically infected HFAs were treated with a minimally effective amount of goat anti-AXL or non-immune goat IgG for 4 days after which cell viability (Figure S4G) and viral genome levels were assessed. Compared to cells treated with non-immune IgG, there was a 40% reduction in genomic RNA levels in the anti-AXL-treated cells suggesting that continuous viral spread, mediated at least in part for AXL receptors, is an important aspect of persistence (Figure 2D).

### 3.4. ZIKV Replicates in HFAs Despite a Robust Antiviral Response

**Figure 3:**
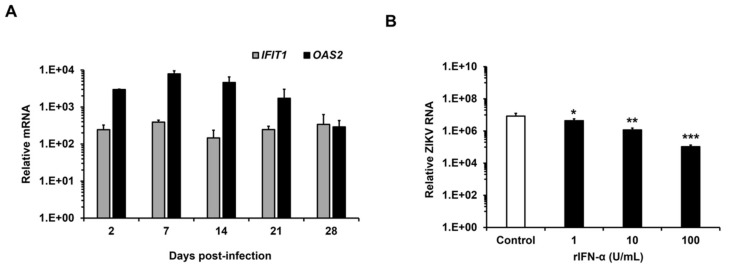

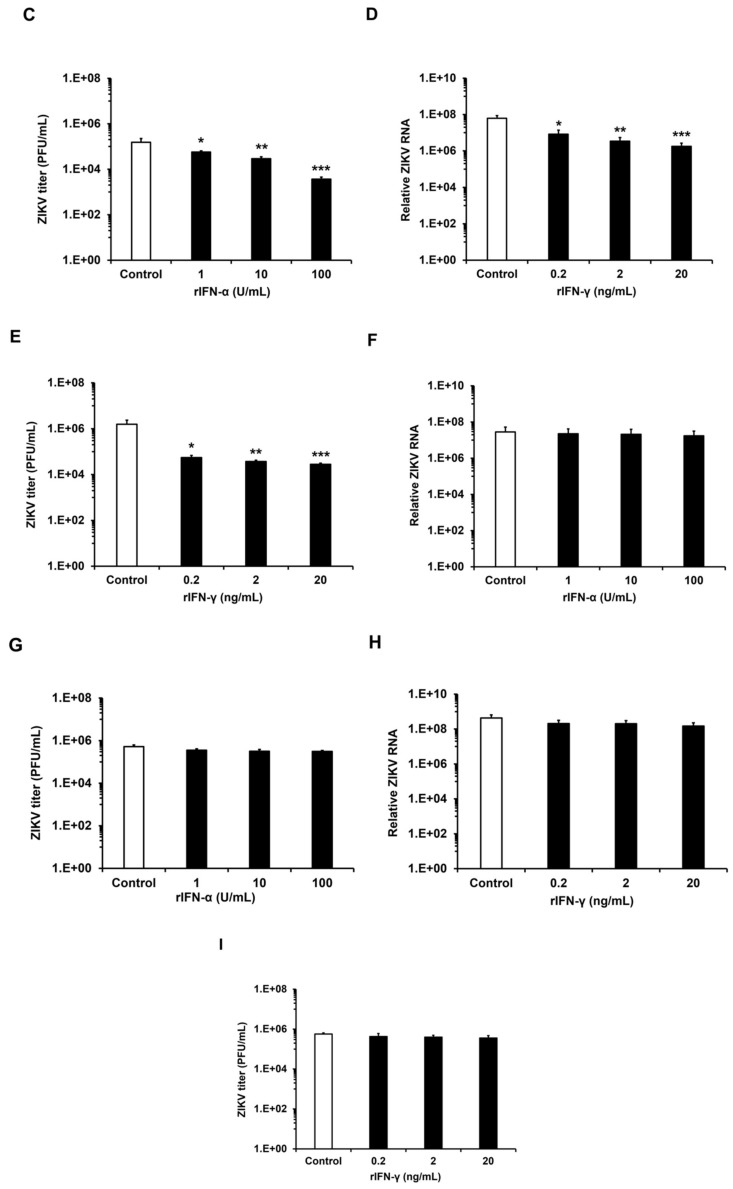
(**A**) Human recombinant IFN treatment and innate immune gene activation in persistently infected HFAs. Relative levels of IFIT1 and OAS transcripts (compared to actin mRNA) in chronically infected HFAs were determined by qRT-PCR. The average values obtained from two experiments using two independent donors are shown. (**B**–**E**) HFAs were treated before (12 and 0 h) or (**F**–**I**) after (24 and 48 h) ZIKV infection (MOI = 0.3) with the indicated amounts of IFN-α and γ. Two days later, supernatants and cell lysates were collected for viral determination by plaque assays (**C**,**E**,**G**,**I**) and viral genome quantitation by qRT-PCR (**B**,**D**,**F**,**H**) respectively. The average values obtained from three experiments using three independent donors are shown. Error bars represent standard error of the mean. * *p* 0.05, ** p 0.01, *** *p* 0.001 (Student’s *t*-test).

We next assessed the immune activation status of the persistently infected HFAs by measuring mRNA levels for 12 IFN-stimulated genes [30]. Quantitation of mRNAs from two representative genes, interferon-induced protein with tetratricopeptide repeats 1 (*IFIT1*) and 2′-5′-oligoadenylate synthetase 2 (*OAS2*) are shown in Figure 3A. Data for the other IFN-stimulated genes are shown in the supplementary information section (Figure S5A–L). Of note, expression of IFN-stimulated genes was high in the chronically infected cells indicating that ZIKV can thrive in HFAs despite a robust antiviral response.

To determine whether IFN treatment can inhibit ZIKV replication in HFAs, cells were treated with recombinant human IFN-α and IFN-γ before or after viral infection. A significant reduction of viral replication and titers was observed in HFAs pre-treated with both IFNs (Figure 3B–E). Conversely, when IFN was added after establishment of the infection, no decrease in ZIKV genome and titers were observed (Figure 3F–I). IFNs concentrations used in these assays did not affect the HFAs viability (Figure S5M,N). Taken together, these data reveal that despite a strong IFN response, ZIKV can establish persistent infection of HFAs. These findings are consistent with our recent results showing that ZIKV blocks IFN signaling [4].

### 3.5. Resistance to Apoptosis Contributes to Persistent Infection of HFAs

**Figure 4:**
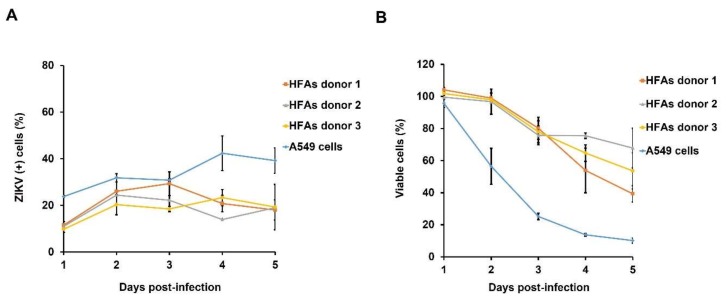

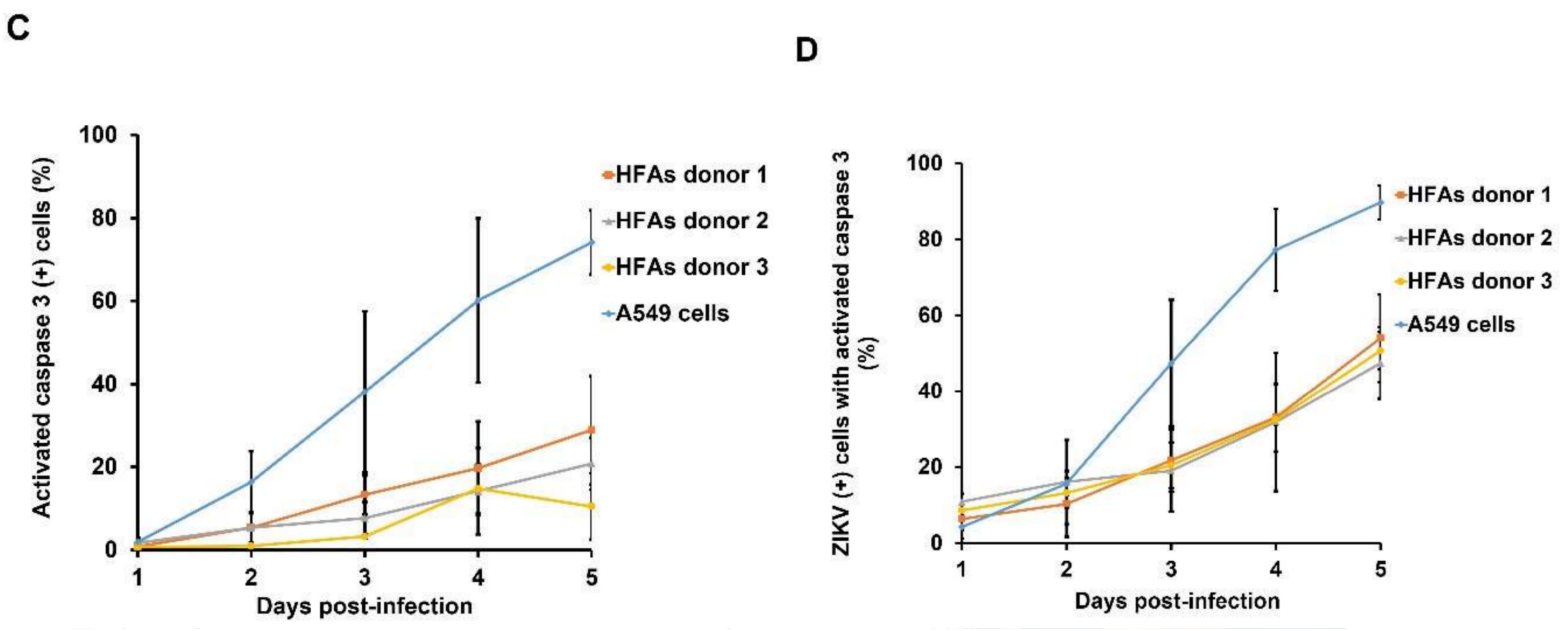
Apoptosis is delayed in ZIKV-infected HFAs. HFAs were infected with ZIKV (MOI = 3) and apoptosis determined by flow cytometry at indicated time points. (**A**) The percentages of HFAs and A549 cells expressing ZIKV envelope protein were determined after 1–5 days post-infection. (**B**) The percentages of viable HFAs and A459 cells remaining after 1–5 days post-infection with ZIKV were determined using an automated cell counter. (**C**) The percentages of HFAs and A549 cells with active caspase-3 are shown. (**D**) The percentages of cells with active caspase-3 among ZIKV-infected HFAs and A549 cells are shown. Values are expressed as the mean of three independent experiments. Error bars represent standard error of the mean.

ZIKV infection has been reported to induce apoptosis in different cell types from human brain [9,31,32]. This phenomenon represents a straightforward mechanism for the observed neuropathology in developing fetuses infected with ZIKV [33]. To determine if ZIKV infection results in dramatic loss of HFAs due to apoptosis, we used flow cytometry to monitor levels of infection and caspase-3 activation over a 5-day infection period (MOI = 3). Data in Figure 4A show that while there was donor-specific variability in the susceptibility of HFAs, by day 5, ~20% of the HFAs were productively infected. A549 cells, which are highly sensitive to ZIKV-induced cytopathic effect, were used as a positive control. Approximately 90% of the A549 cells had died off 5 days post-infection with ZIKV (Figure 4B). In contrast, a much larger proportion of the infected HFAs cultures were still alive on day 5 and the majority of these cells (~70%) were not apoptotic (Figure 4B–C).

To determine whether the cell death in HFAs cultures was a direct consequence of ZIKV infection, we quantified caspase-3 activation in cells that were positive for ZIKV antigen. Interestingly, there was a delay in activation of caspase-3 in HFAs following ZIKV infection. While the majority (~90%) of infected-A549 cells were apoptotic by day 5, less than 50% of the infected HFAs exhibited signs of apoptosis (Figure 4D). Representative primary data from the flow cytometry analyses of A549 cells and HFAs are included in the supplementary material (Figure S6). These results indicate that while a pool of HFAs is highly permissive to infection by ZIKV, the cells are comparatively resistant to virus-induced apoptosis. Together, these data are consistent with a scenario in which HFAs can serve as a reservoir for ZIKV persistence in the fetal brain.

### 3.6. Persistent ZIKV Infection Has A Dramatic Effect on HFA Transcription

**Figure 5:**
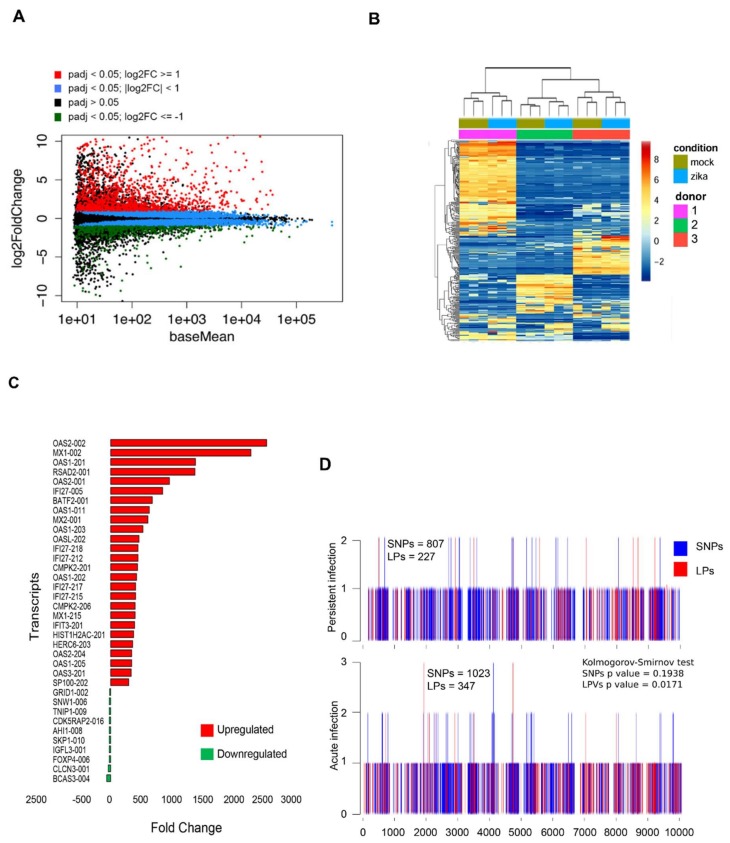
(**A**) Analysis of transcripts differentially expressed in HFAs persistently infected (28 days) with ZIKV. MA plots representing transcripts that were deregulated in cells persistently infected with ZIKV are shown. Red dots represent transcripts that were significantly upregulated with a fold change 2; green dots represent transcripts that were significantly downregulated with a fold change –2; blue dots represent genes that were significantly deregulated with a fold change 2; black dots represent genes not differentially expressed. (**B**) Hierarchical clustering identified two major clusters of differentially expressed genes in persistently infected HFAs (28 days), with relatively consistent expression across samples (i.e., consistently up or downregulated in ZIKV-infected or mock samples). *Z*-scores of 400 differentially expressed genes exhibiting the largest variance between mock and ZIKV-infected samples are plotted. (**C**) The fold-change and the corrected P value of the top up- and downregulated transcripts are presented for persistently infected HFAs (28 days). (**D**) Mutations are not accumulated in the ZIKV genome after persistent infection. Single nucleotide polymorphisms (SNPs) and length polymorphisms (LPs) were determined in the ZIKV genome in acute (2 days) and persistent (28 days) infection of HFAs using the software Vphaser-2. Kolmogorov–Smirnov test was applied on the frequency of each type of mutations consolidated in segments of 500 nt.

To determine how chronic ZIKV infection affects the transcriptome of HFAs, we performed RNAseq analyses of cells that were infected for 28 days. Most of the genes that were deregulated by viral infection (|fold change| 2; FDR 0.05) were upregulated (Figure 5A,B). In total, 722 transcripts were upregulated, while 81 transcripts were downregulated (Table S2) by chronic ZIKV infection. Strikingly, many genes involved in the antiviral response were significantly upregulated (Figure 5C). For example, expression of *OAS2*, the most upregulated gene during persistent infection, increased 2000-fold in response to ZIKV infection. Among the upregulated genes in persistently infected HFAs, the average increase in expression level was 49-fold. Of note, histone cluster 1 H2A family member c (*HIST1H2AC*) which encodes a histone protein linked to cell differentiation [34], was upregulated 380-fold in persistently infected cells (Figure 5C). One of the genes whose expression was most suppressed by ZIKV in persistently infected HFAs encodes the forkhead box protein P4 (*FOXP4*), a transcription factor involved in development of the central nervous system [35]. The biological relevance of these intriguing findings will require extensive further investigation.

We also conducted gene ontology analysis (using the Cytoscape plug-in clueGO) [26] to study gene deregulation during persistent ZIKV infection of HFAs. In persistently infected cells, extensive deregulation of genes that affect morphogenesis of epithelium, cell-substrate adherens junction assembly and focal adhesion assembly were observed (Table S2). The clueGO analysis of upregulated genes identified 10 top terms exclusively related with antiviral defense for persistently infected cells (Figure S7A).

Finally, to validate data from the RNAseq transcriptome profiles, we assessed expression of a subset of the most differentially expressed genes in persistently infected HFAs using qRT-PCR. The expression patterns of *CMPK2*, *BATF2*, *MX1*, *MX2*, *RSAD2* (viperin), *IFI27* and *OAS2* paralleled those obtained from the RNAseq dataset (Figure S7B).

### 3.7. No Significant Mutations Are Accumulated in the ZIKV Genome After Persistence

We determined the mutation burden (SNPs an LPs) along the virus genome using the software Vphaser-2 [27]. Both, SNPs and LPs were slightly more abundant in acute infection (1,023 vs. 807 and 347 vs. 227, respectively). We found that the number of mutations in such transepts was significantly different for LPs (*p* = 0.0171), but not for SNPs. In other words, more length polymorphisms accumulated during acute infection than during persistent infection (Figure 5D).

## 4. Discussion

While a number of animal and human studies have addressed the neurotropism of ZIKV [5,15,36,37], many unanswered questions remain regarding the mechanisms of immune evasion and viral adaptation in target brain cells. Following peripheral inoculation, ZIKV can be detected for up to 28 days in the brains of adult mice lacking IFN α/β receptors [5]. Moreover, genomic RNA and infectious virus have been recovered from human fetal brain several months after maternal infection [13,33]. Prior to this study though, the only known human cell type that supports ZIKV infection for up to 28 days are in vitro-derived fetal neural progenitor cells [7]. The significance of this observation in ZIKV biology remains unclear as it is unlikely that neural progenitor cells are abundant enough to serve as an efficient reservoir. Here, we show for the first time that primary HFAs can support persistent and productive infection of ZIKV for at least one month and propose a model for persistent infection of HFAs (Figure S8). Moreover, the use of HFAs *ex vivo* may be useful as a platform for testing and developing compounds that prevent chronic ZIKV infection of the fetal brain.

Compared to acute infection, we observed moderate viral titers that correlate with the comparatively low levels of ZIKV-positive cells in persistently infected HFAs cultures. This could be due to a sustained antiviral response that limits viral replication and spread, resulting in equilibrium between viral persistence and cell survival. The stochastic nature of cellular antiviral response may underlie the ability of ZIKV to spread in HFAs cultures despite a sustained antiviral response. Once the virus initiates infection, it can deploy effective countermeasures against cellular antiviral defense (IFN response) thus enabling it to productively replicate as we [4] and others [38] have recently shown in different cell lines. Nevertheless, robust IFN production and signaling still occurs during flavivirus infection [39]. It is likely that in the absence of viral interference with host anti-viral pathways that the IFN response would start earlier and be even more robust. At the very least, many viruses are able to delay or dampen the IFN response long enough to allow a window of time for replication and egress but they are not able to completely block this critical host anti-viral pathway.

We observed that administration of IFN prior to infection limits multiplication of ZIKV in HFAs. Similar findings were reported for primary mouse postnatal astrocytes infected with another neurotropic flavivirus, tick-borne encephalitis virus [11]. It is tempting to speculate that a sustained innate immune response in the brain contributes at least in part to the neurological defects associated with congenital Zika infection [40] by affecting cellular migration and differentiation.

Acute infection as well as viral spread in persistently infected HFAs involves TIM/TAM receptors. This fits with previous data showing that in the developing human brain cortex, the TAM family protein AXL is highly expressed in astrocytes [14]. More recently, during acute infection, AXL-mediated ZIKV entry was reported in in vitro-stem cell-derived astrocytes [15] and primary human adult astrocytes [16]. Furthermore, the observation that antibody- or drug-mediated blocking of AXL significantly reduces infection of HFAs is consistent with findings from others [15,16] supporting a role for AXL as an entry receptor on astrocytes. Although it was speculated that TIM/TAM family receptors may be important for long-term ZIKV infection of astrocytes in the fetal brain, here, using AXL receptor inhibition, we provide the first evidence that this is indeed the case. Recently, we showed that AXL is also important for ZIKV infection of human Sertoli cells [23]. Conversely, infection of in vitro-derived fetal neural progenitor cells appears to be AXL-independent [41]. This suggests that ZIKV can use different entry receptors depending upon cell type.

Several flaviviruses including West Nile virus [42], tick-borne encephalitis virus [43] and dengue virus [44] have been reported to induce apoptosis following infection of human brain tissue. Similarly, extensive cell death has been observed during ZIKV infection of human fetal neural progenitors-derived cerebral organoids [31], tissue slices from developing brain [15,45] and neurons from fetal brain tissue [33]. While our studies indicate that some virus-induced apoptosis does occur in HFAs, the process of cell death is significantly less than the levels observed in A549 cells within the same period of time. If this situation occurs *in vivo*, it could explain the observed prolonged virus shedding and persistence of ZIKV in the fetal brain.

Analyses of gene expression changes in persistently infected HFAs revealed that multiple transcripts involved in the IFN response were highly upregulated. Our results are consistent with PCR array data from a recent study of human primary astrocytes with acute infection by ZIKV [46]. In contrast, acute infection of human primary fibroblasts with ZIKV revealed that the virus induces specific activation of inflammasome components such as *AIM2* and interleukin-1β (*IL-1β*) [47]. We also observed downregulation of a gene *FOXP4* [35] that is associated with brain development, during persistent infection of HFAs.

Mutations acquired during the course of chronic infection can produce attenuated forms of a virus that favor persistent infections [48,49]. However, we did not observe any compensatory changes in genomic RNA of ZIKV isolated from persistently infected HFAs. Some length polymorphisms did accumulate during acute infection rather than persistent infection though. Consistent with our data, a recent study of MR766 ZIKV replication suggests that the viral genome does not tolerate a high degree of variation [50]. In contrast, another report revealed a significant increase of MR766 ZIKV infectivity in the human monocytic leukemia U937 cell line after continuous culture for 3 months that resulted in the development of distinct sets of stable mutations associated with amino acid changes [51].

## 5. Conclusions

Here, we show that HFAs, the most abundant cell type in brain tissue, can support chronic ZIKV infection and continuous viral shedding for at least one month. Our findings suggest that resistance to apoptosis and the antiviral response kinetics displayed by HFAs may facilitate their role as a viral reservoir in the fetal brain. Finally, a number of virus-induced changes in host gene expression in HFAs provide additional clues as to how neurodevelopmental processes may be affected by ZIKV.

## Supplementary Materials

The following are available online at http://www.mdpi.com/1999-4915/10/11/646/s1. **Figure S1**. Measurement of GFAP and AXL expression in HFAs by flow cytometry. Release of ZIKV NS1 by HFAs. (**A**) The percentage of HFAs expressing the astrocyte marker GFAP were assessed by flow cytometry after two days in culture. U373, an astrocytoma cell line that is known to express GFAP, was used as a positive control. (**B**) Levels of ZIKV NS1 in HFAs supernatants 1–4 days post-infection were measured using an in-house ELISA. The percentages of AXL-positive HFAs (**C**) and mean fluorescence (**D**) of AXL signal from three donors were determined by flow cytometry. A549 cells were used as a positive control. **Figure S2**. Effect of TIM/TAM inhibitors on ZIKV infection of HFAs. High-content imaging was used to analyze the effect of TIM/TAM receptor inhibition on infection of HFAs treated with three different concentrations of (**A**) Duramycin (TIM1 inhibitor) or Control (HCl diluent) and (**B**) R428 (AXL inhibitor) or Control (DMSO diluent). Representative images are shown. **Figure S3**. Effect of TIM/TAM receptor inhibitors on ZIKV infection, viral replication and cell viability in A549 cells and on cell viability of HFAs. HFAs were treated with (**A**) duramycin or (**B**) R428 for 24 h at indicated concentrations and cell viability was determined using the CellTiter-Glo Luminescent Cell Viability Assay. (**C**,**D**) A549 cells were either infected with ZIKV pre-incubated with duramycin for 1 h or treated with R428 for 1h followed by infection with ZIKV (MOI = 0.5). One day later cells were processed for indirect immunofluorescence or processed for total RNA extraction. Quantitation of A549 cells positive for ZIKV envelope protein was performed using the Operetta High Content Imaging System. Panels E and F show quantification of ZIKV replication by qRT-PCR. Cell viability was determined using the CellTiter-Glo Luminescent Cell Viability Assay in A549 cells treated with (**G**) duramycin or (**H**) R428 for 24 h at indicated concentrations. (**I**) A549 cells were pre-incubated with anti-AXL blocking antibody for 1 h followed by ZIKV infection (MOI = 10). After 24 h, cells were collected and ZIKV RNA was quantified by qRT-PCR. Values are expressed as the mean of three independent experiments. Error bars represent standard error of the mean. * *p* 0.05, ** *p* 0.01, *** *p* 0.001 (Student’s *t*-test). **Figure S4**. GFAP expression and persistent infection of HFAs. Representative confocal images of GFAP and ZIKV antigen staining in HFAs donor 2 (**A**) and HFAs donor 3 (**B**) are shown. (**C**) The percentages of GFAP-positive HFAs after four weeks was determined by flow cytometry. The average values (± standard error of the mean) obtained from experiments using three HFAs donors are shown. (**D**–**F**) Viral persistence as demonstrated by continuous viral shedding in HFAs from three donors. HFAs were infected with MR766 ZIKV at MOI of 3 (**D**) and PRVABC-59 ZIKV at MOI of 3 (**E**) and 0.3 (**F**). (**G**) HFAs were treated with a minimal amount of anti-AXL antibody (4 μg/mL) or non-immune goat IgG for 4 days after two weeks in cell culture and cell viability was determined using the CellTiter-Glo Luminescent Cell Viability assay. **Figure S5**. Activation of IFN-stimulated genes in persistently infected HFAs. (**A**–**L**) The transcript levels of *IFIT1, CXCL10*, *CXCL5*, *CXCL9*, *CXCL11*, *MX1*, *MX2*, *IFI27*, *CMPK2*, *OASL*, *OAS2* and *IFN-β* from mock or ZIKV-infected HFAs were determined by qRT-PCR over a time course of 28 days. (**M**,**N**) After HFAs were treated with human recombinant IFN-α or IFN-γ for 2 days, cell viability was determined using the CellTiter-Glo Luminescent Cell Viability assay. **Figure S6**. Representative gating strategies for ZIKV-infected A549 cells and HFAs. Cells were infected with ZIKV (MOI = 3) and 5 days later, were harvested and processed for flow cytometry. Apoptotic cells (based on PE-Texas Red fluorescence) or ZIKV infection (based on Alexa Fluor 405 fluorescence) were assessed. The percentages of A549 cells and HFAs that were double positive for ZIKV antigen and activated caspase-3 at indicated times during the 5-day infection period are shown. **Figure S7**. Comparative analysis of differentially expressed host transcripts and validation of RNASeq data by qRT-PCR in chronically infected HFAs. (**A**) Top ten deregulated terms derived with upregulated genes in persistently infected cells (according to Bonferroni-corrected *p* values). (**B**) Relative transcript levels of *CMPK2*, *BATF2*, *MX1*, *MX2*, *RSAD2* (viperin), *IFI27* and *OAS2* were determined at four weeks post-infection. Values are expressed as the mean of three independent experiments. Error bars represent standard error of the mean. **Figure S8**. Model depicting ZIKV persistence infection in HFAs. HFAs are highly permissive to ZIKV infection mediated by TIM/TAM receptors. After an acute period characterized by moderate apoptosis in infected cells, a persistent infection is stablished. During persistence, viral replication is decreased but the virus continues to spread from cell to cell despite a sustained antiviral response. **Table S1**. Primer sets for qRT-PCR used in the study. **Table S2**. Sheet 1. Differentially expressed genes as detected by the Kallisto/Sleuth pipeline in persistently infected cells. Kallisto was run with default parameters. During DESeq2 analysis, two factors were defined, one for donor (including levels 1, 4 and 5) and another for condition (including levels of Zika and control), and the following design was defined for analysis: sleuth_prep (s2c, ~ condition + patient, target_mapping = t2g). Results with a FDR 0.05 and a fold-change 2 were considered differentially expressed, and finally were annotated with BioMart. Upregulated genes are in black font and downregulated are in blue font. Sheet 2. Gene ontology analysis using clueGO for transcripts that were upregulated during persistent infection of HFAs with ZIKV. Analyses were run with default parameters. Only overrepresented terms that had a corrected *p* value 0.05 are shown.
