# Supplementary material for: Human Fetal Astrocytes Infected with Zika Virus Exhibit Delayed Apoptosis and Resistance to Interferon: Implications for Persistence
Source: Viruses. 2018 Nov 17;10(11):646. doi: 10.3390/v10110646 (PMC6266559; doi:10.3390/v10110646)
Supplement: Supplementary file 1 [file viruses-10-00646-s001.zip › viruses-377889-supplementary/Limonta et al, Supplementary figures, Proofreading VIRUSES Nov16,2018.pdf]

Figure S1

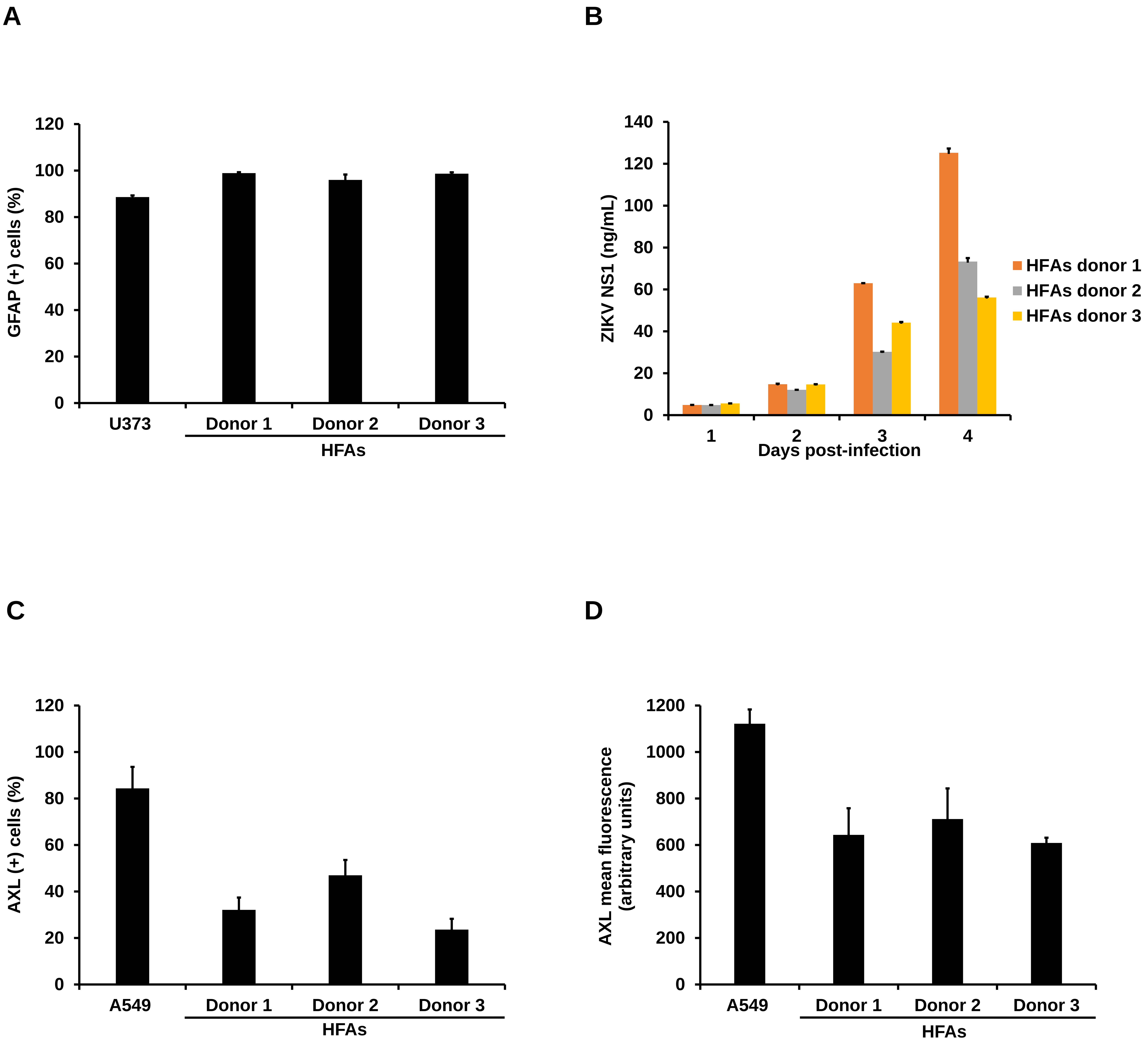
A
120
100
80
60
40
20
0
GFAP (+) cells (%)
U373
Donor 1
Donor 2
Donor 3
HFAs
B
140
120
100
80
60
40
20
0
ZIKV NS1 (ng/mL)
1
2
3
4
Days post-infection
HFAs donor 1
HFAs donor 2
HFAs donor 3
C
120
100
80
60
40
20
0
AXL (+) cells (%)
A549
Donor 1
Donor 2
Donor 3
HFAs
D
1200
1000
800
600
400
200
0
AXL mean fluorescence
(arbitrary units)
A549
Donor 1
Donor 2
Donor 3
HFAs

Figure S2

A

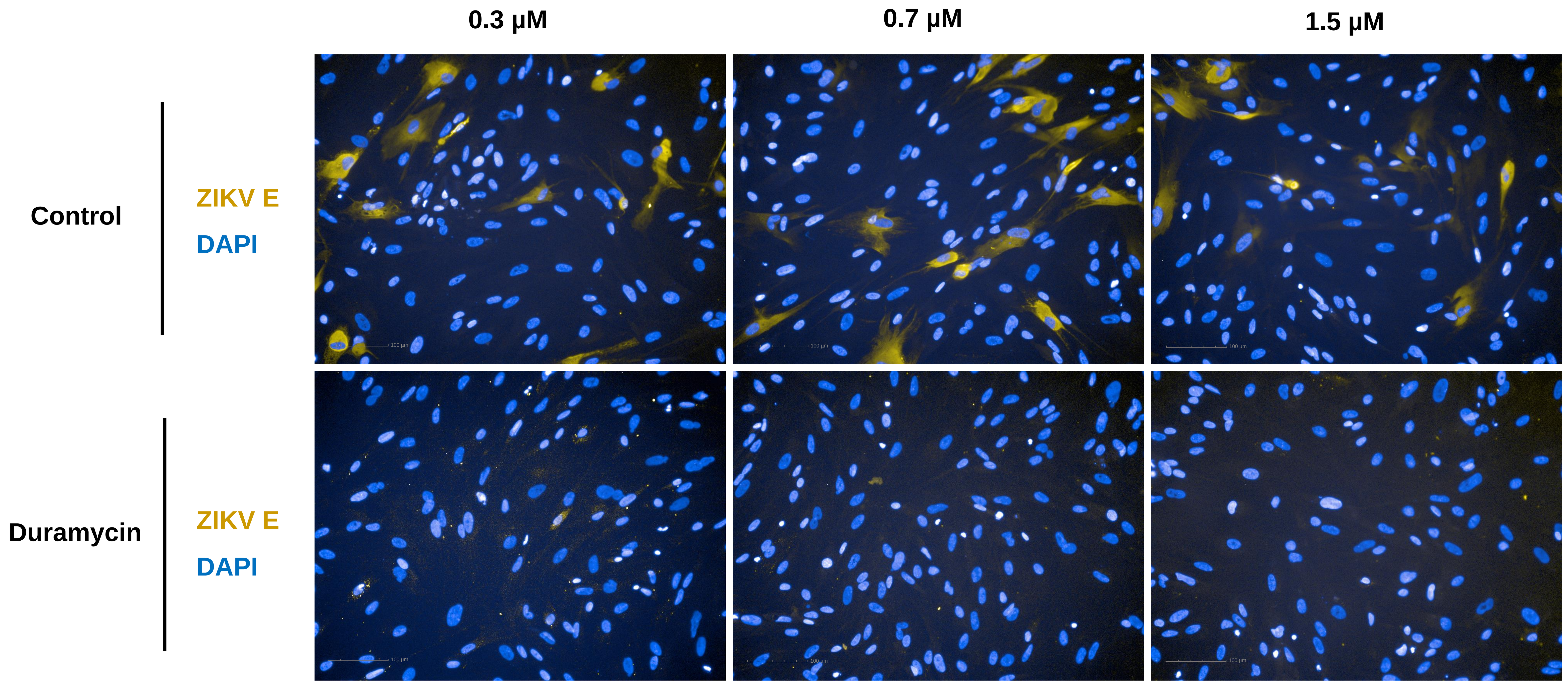
0.3 μM
0.7 μM
1.5 μM
Control
ZIKV E
DAPI
Duramycin
ZIKV E
DAPI

B

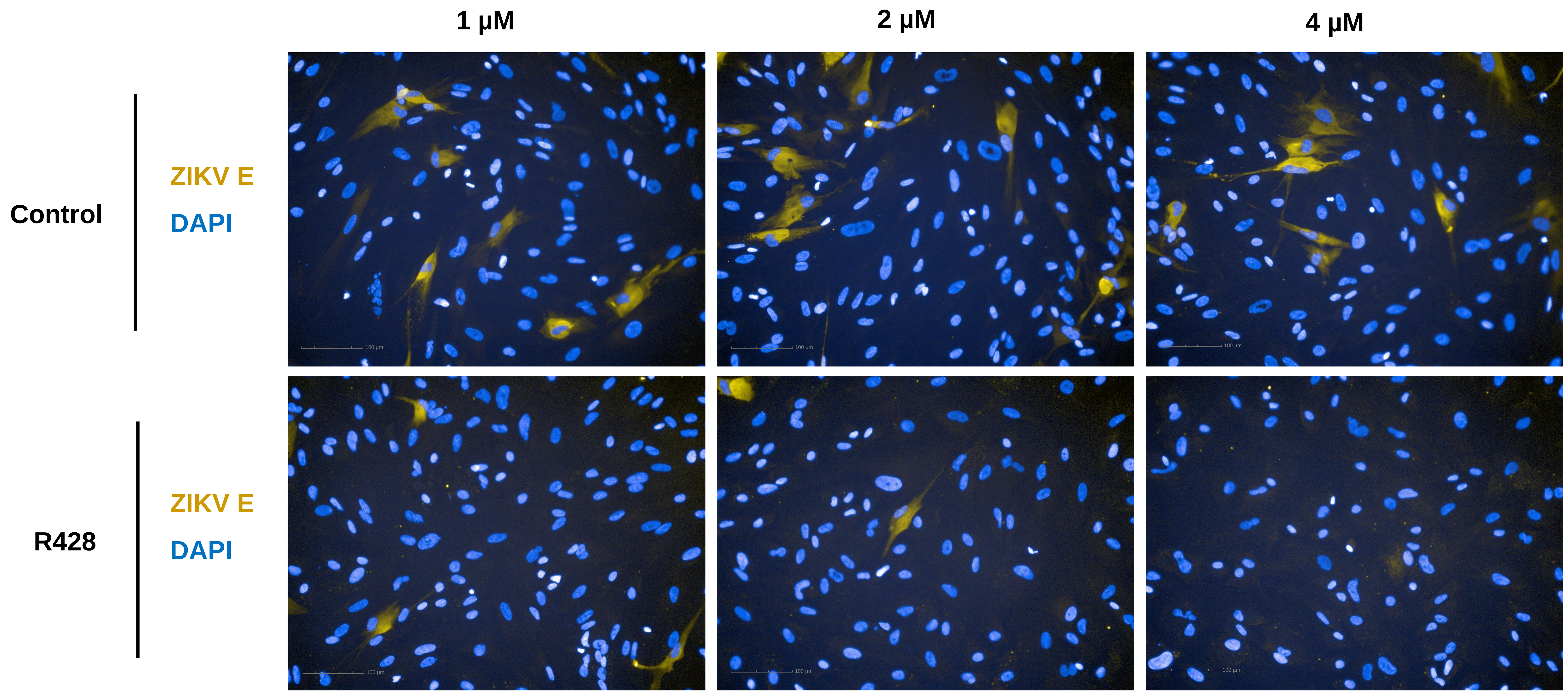
1 μM
2 μM
4 μM
Control
ZIKV E
DAPI
R428
ZIKV E
DAPI

Figure S3

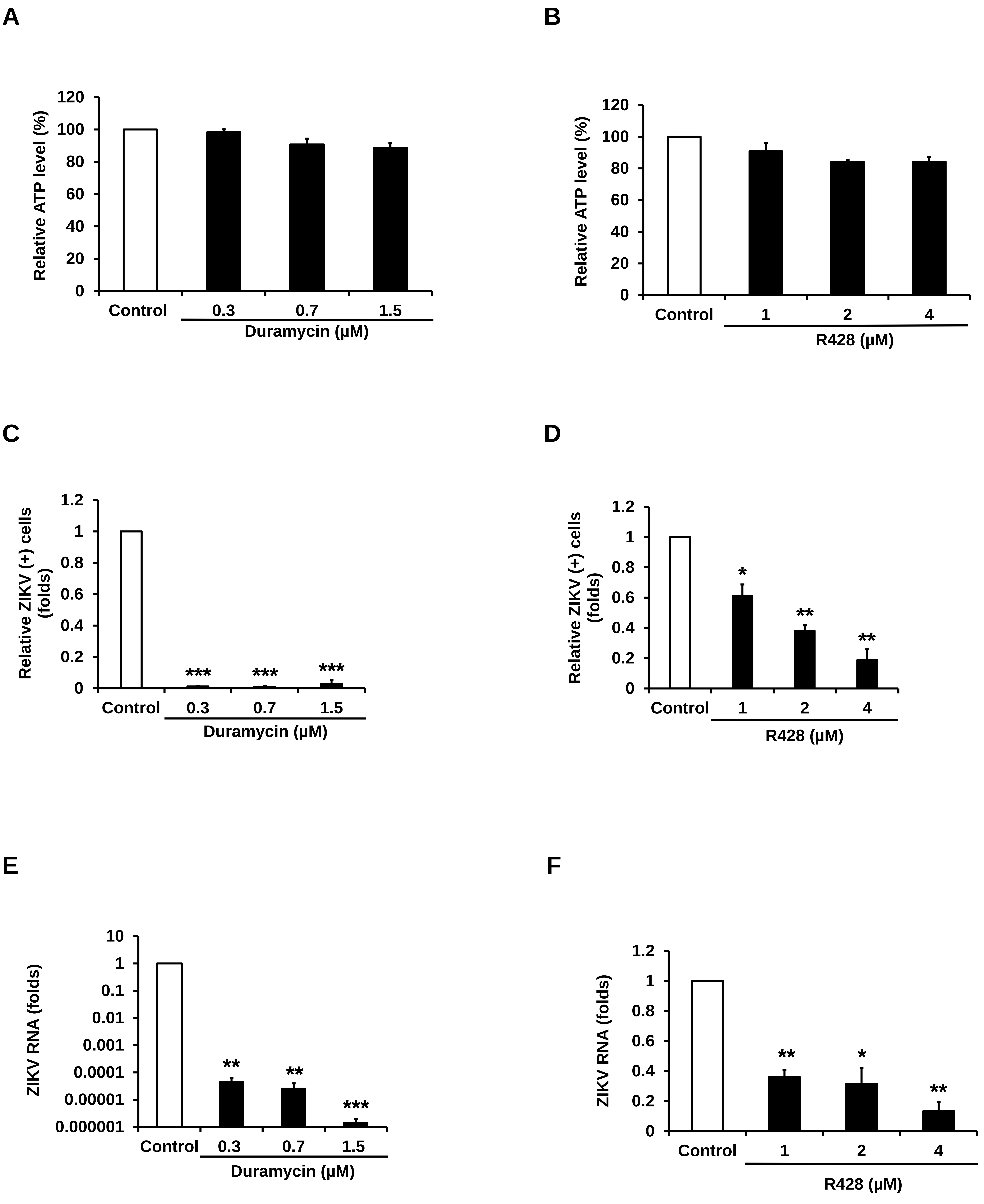

A
B
C
D
E
F
Relative ATP level (%)
120
100
80
60
40
20
0
Control
0.3
0.7
1.5
Duramycin (μM)
1
2
4
R428 (μM)
Relative ZIKV (+) cells (folds)
1.2
1
0.8
0.6
0.4
0.2
*
**
***
ZIKV RNA (folds)
10
0.1
0.01
0.001
0.0001
0.00001
0.000001

Figure S3

G

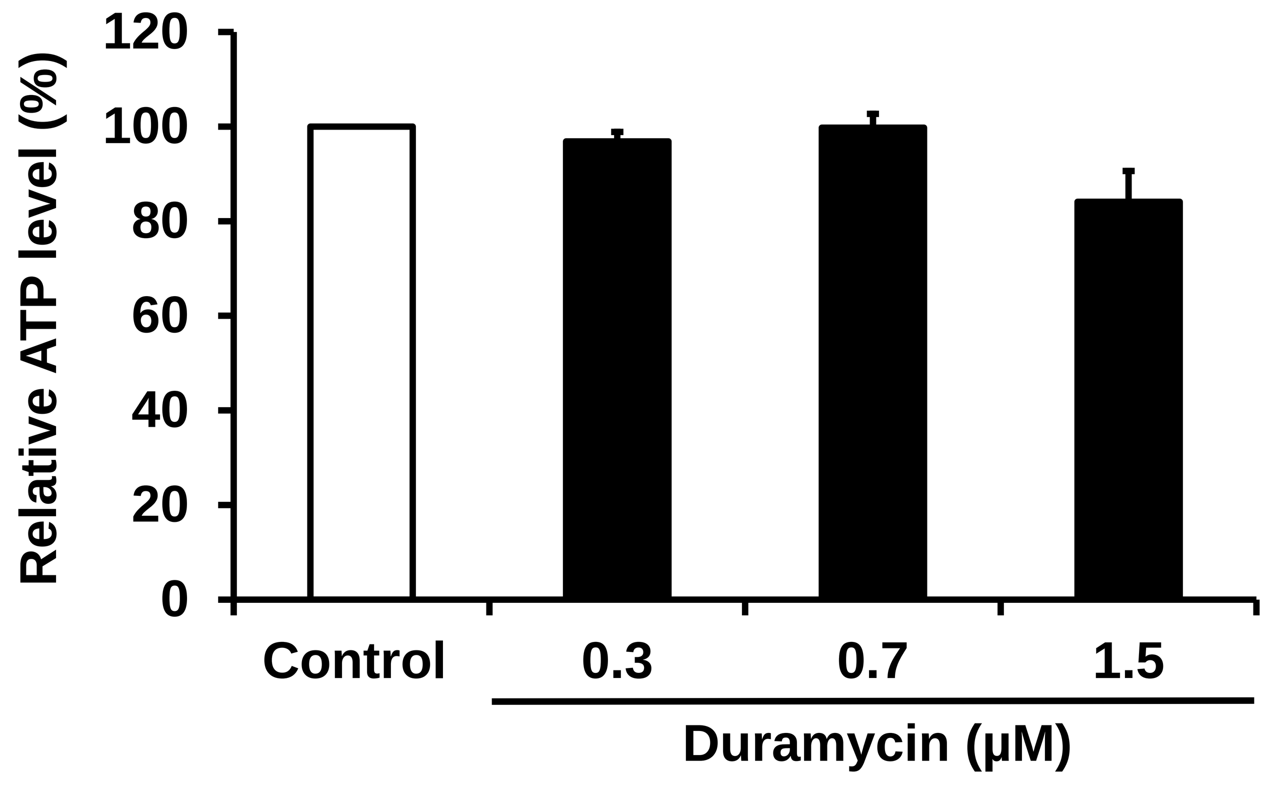

H

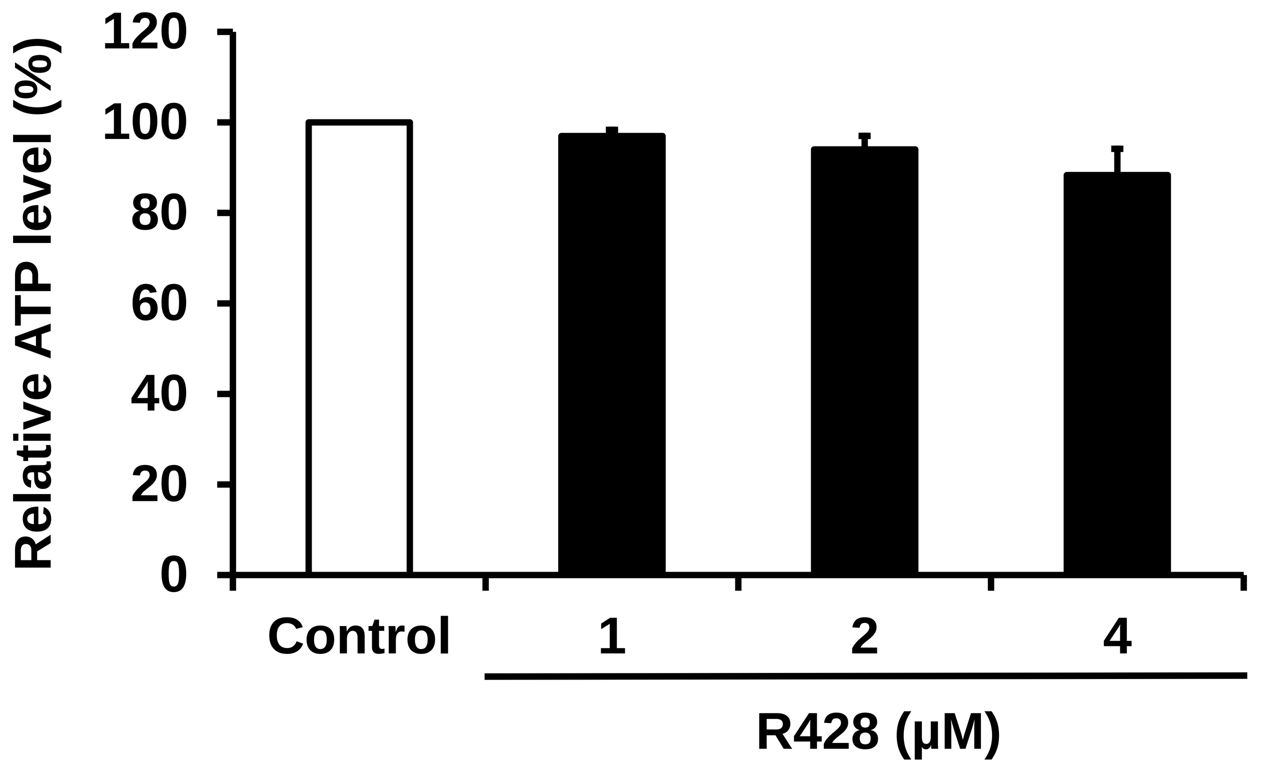

I

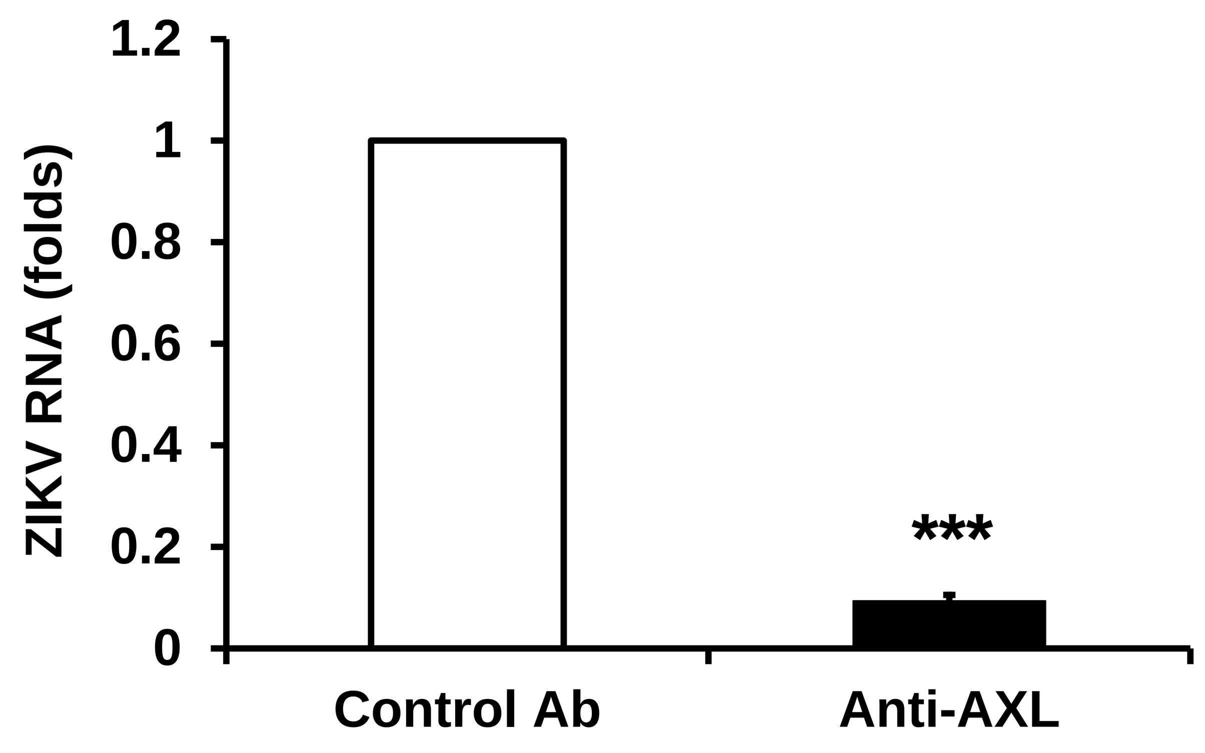

Figure S4

A

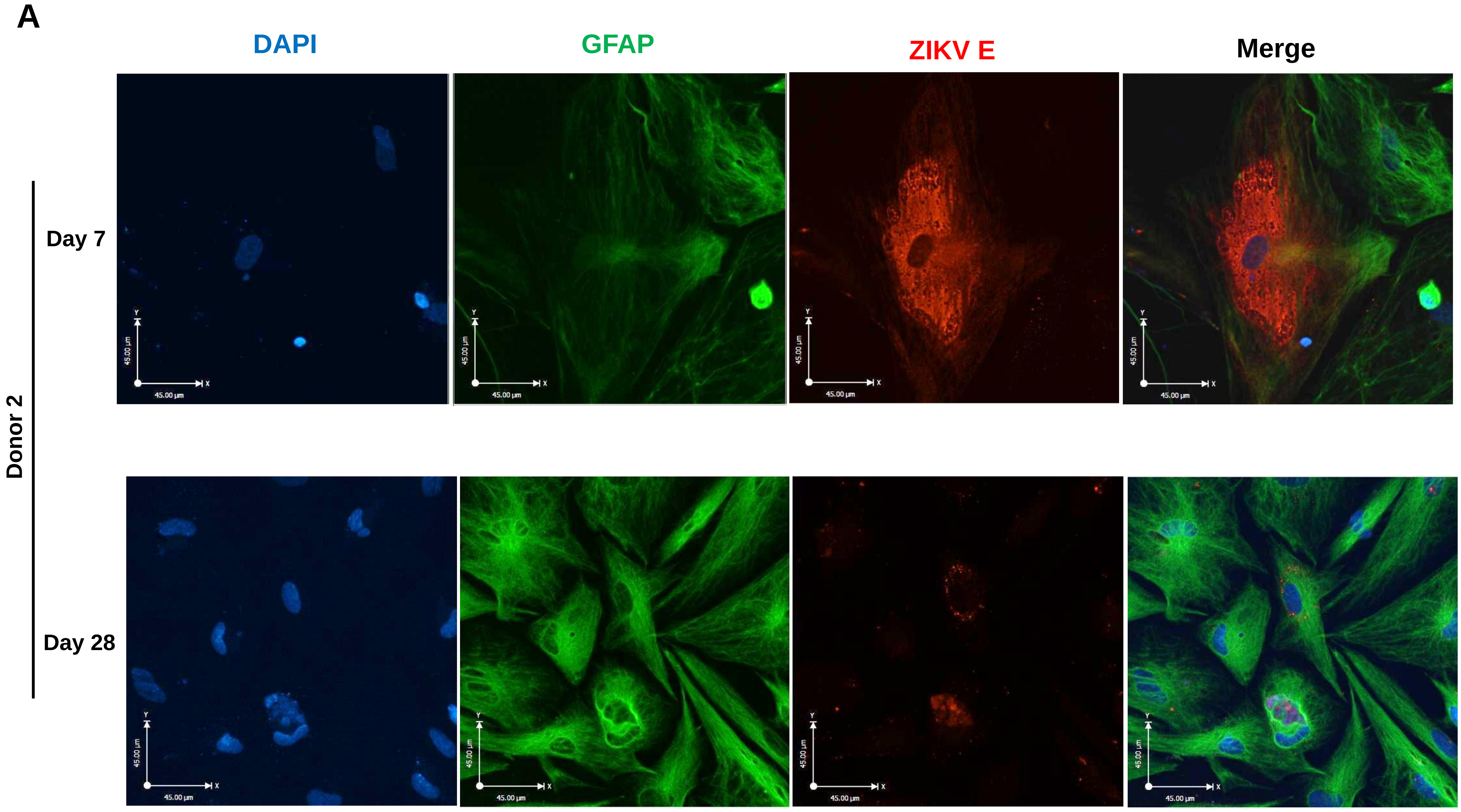
DAPI
GFAP
ZIKV E
Merge
Donor 2
Day 7
Day 28

B

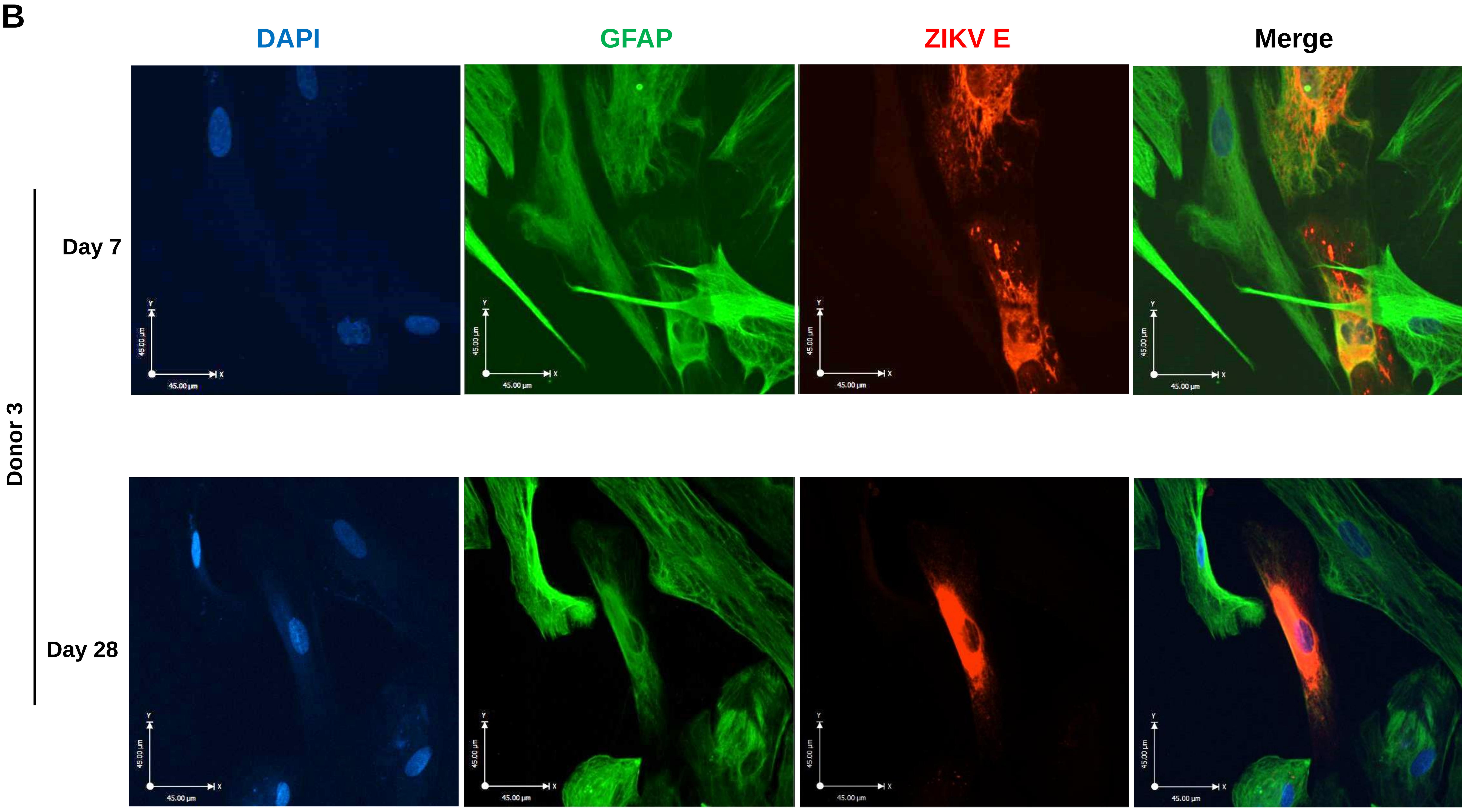
DAPI
GFAP
ZIKV E
Merge
Donor 3
Day 7
Day 28

Figure S4

C

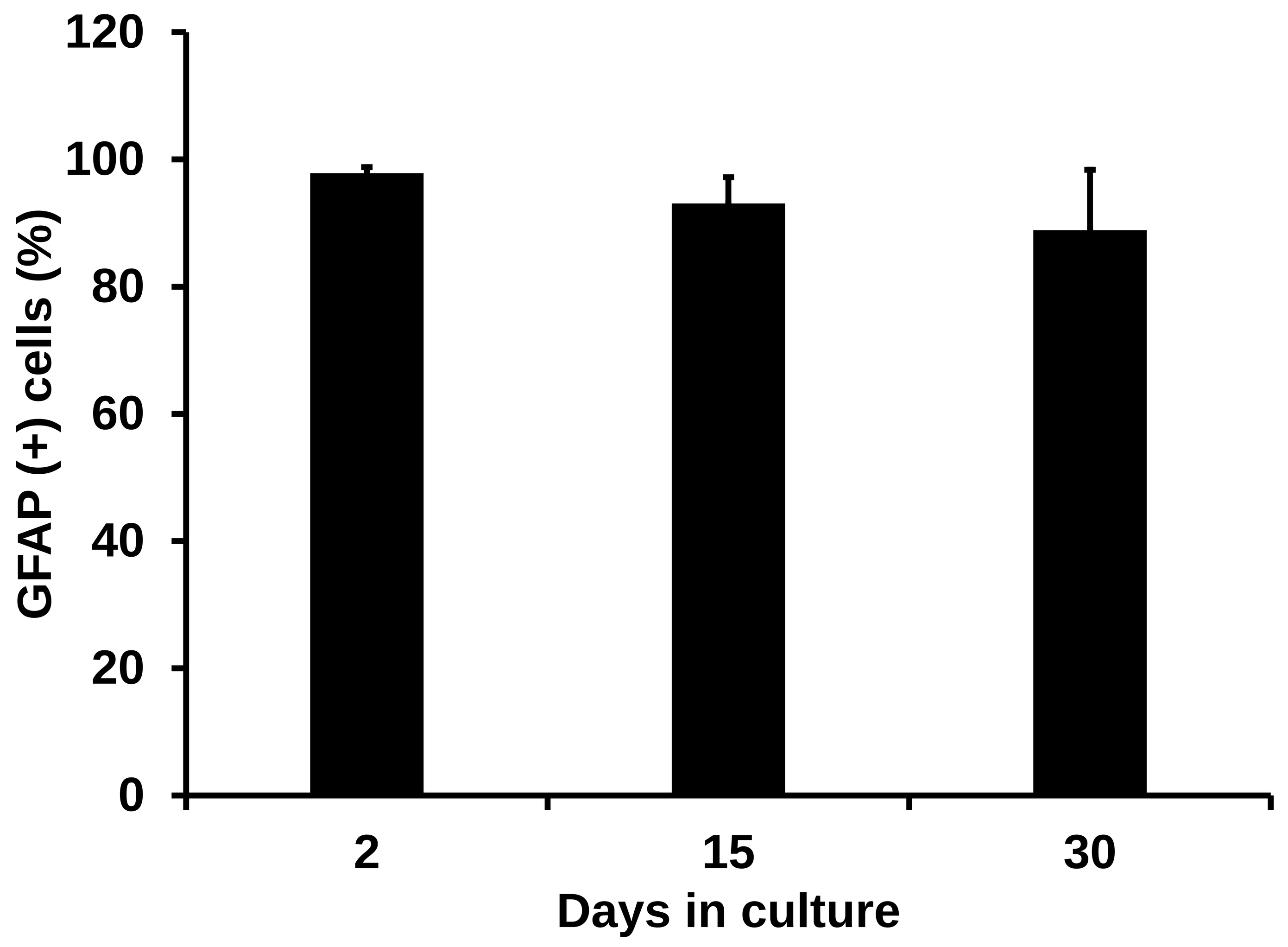

D

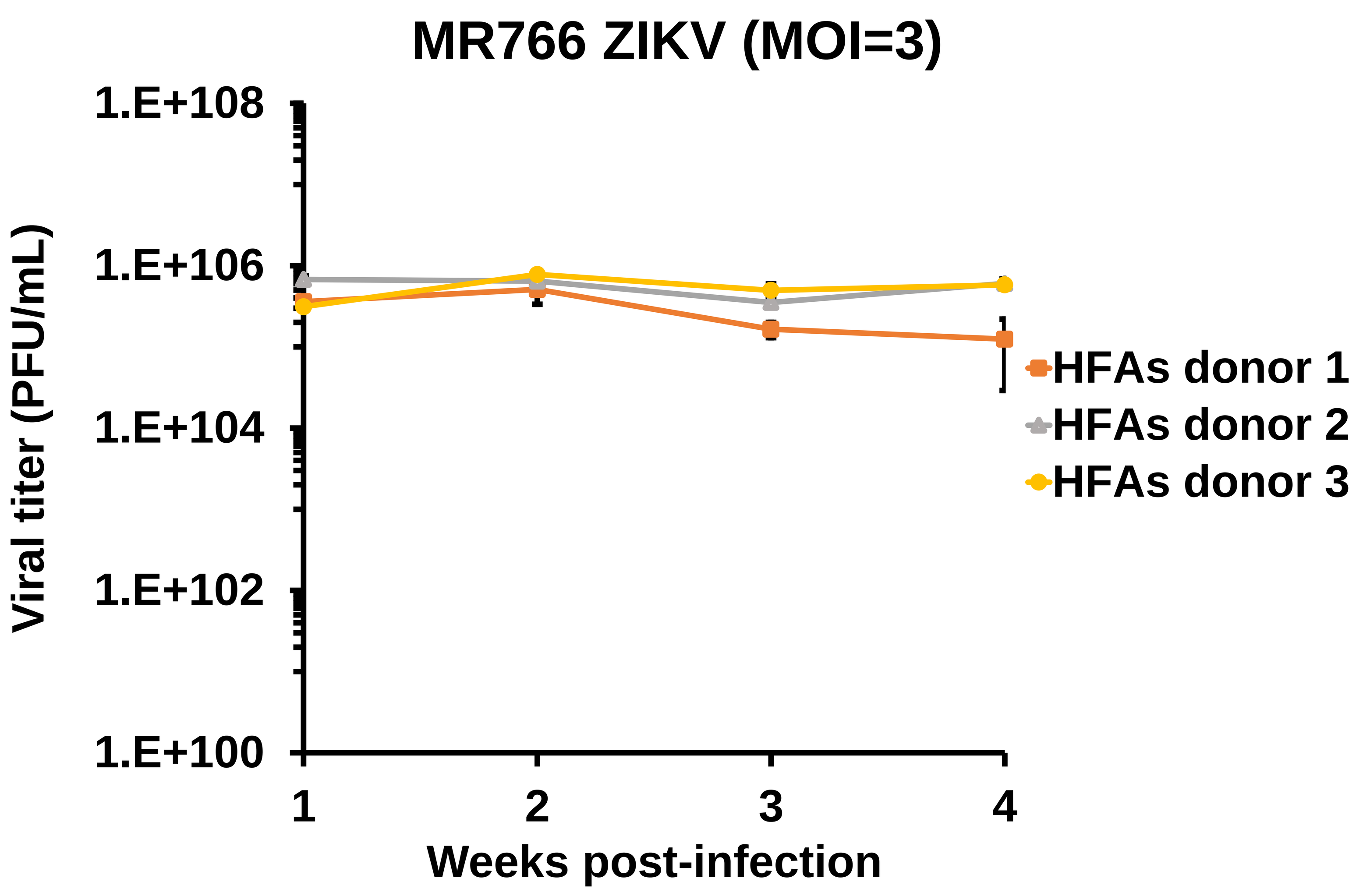

E

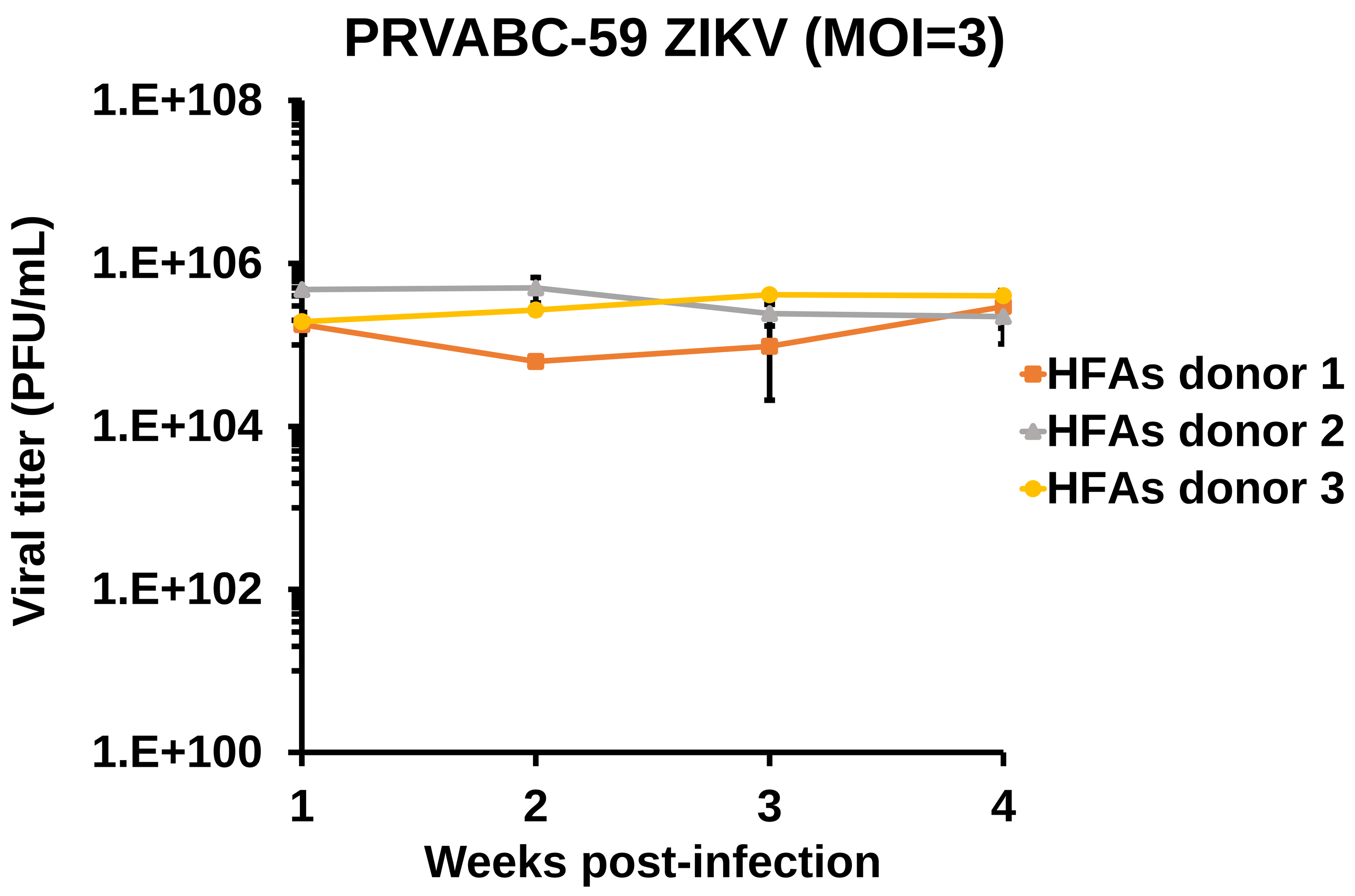

F

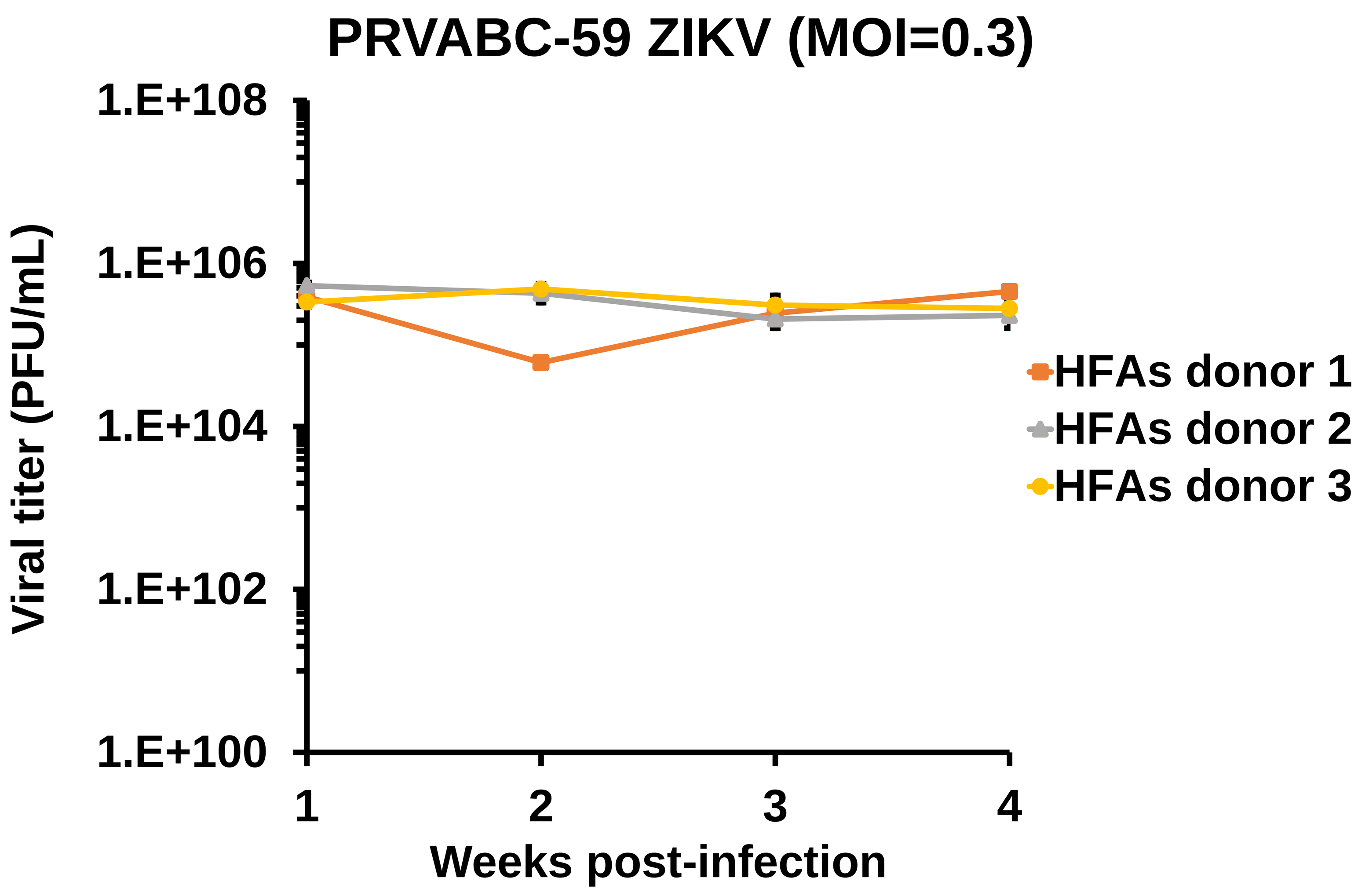

G

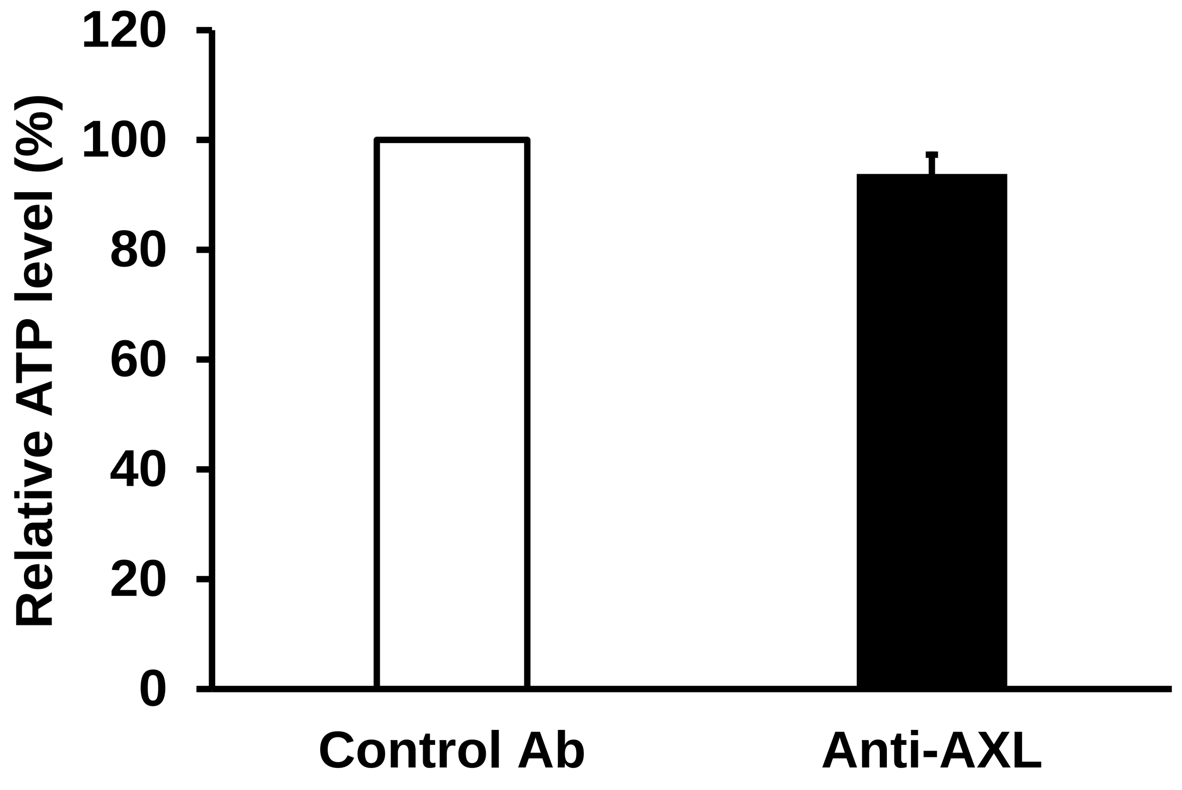

Figure S5

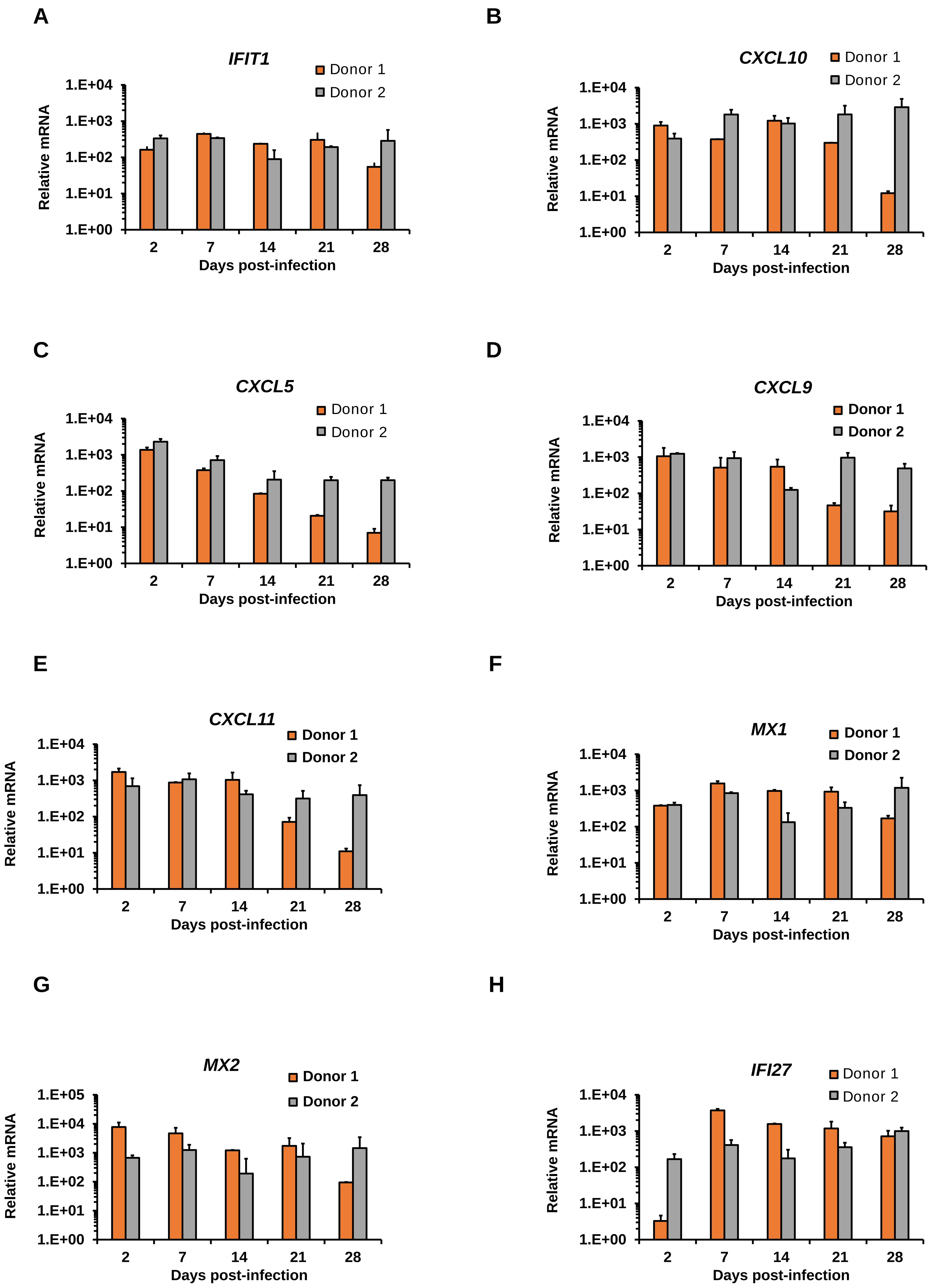
A
IFIT1
Donor 1
Donor 2
Relative mRNA
1.E+04
1.E+03
1.E+02
1.E+01
1.E+00
2
7
14
21
28
Days post-infection
B
CXCL10
C
CXCL5
D
CXCL9
E
CXCL11
F
MX1
G
MX2
1.E+05
H
IFI27

Figure S5

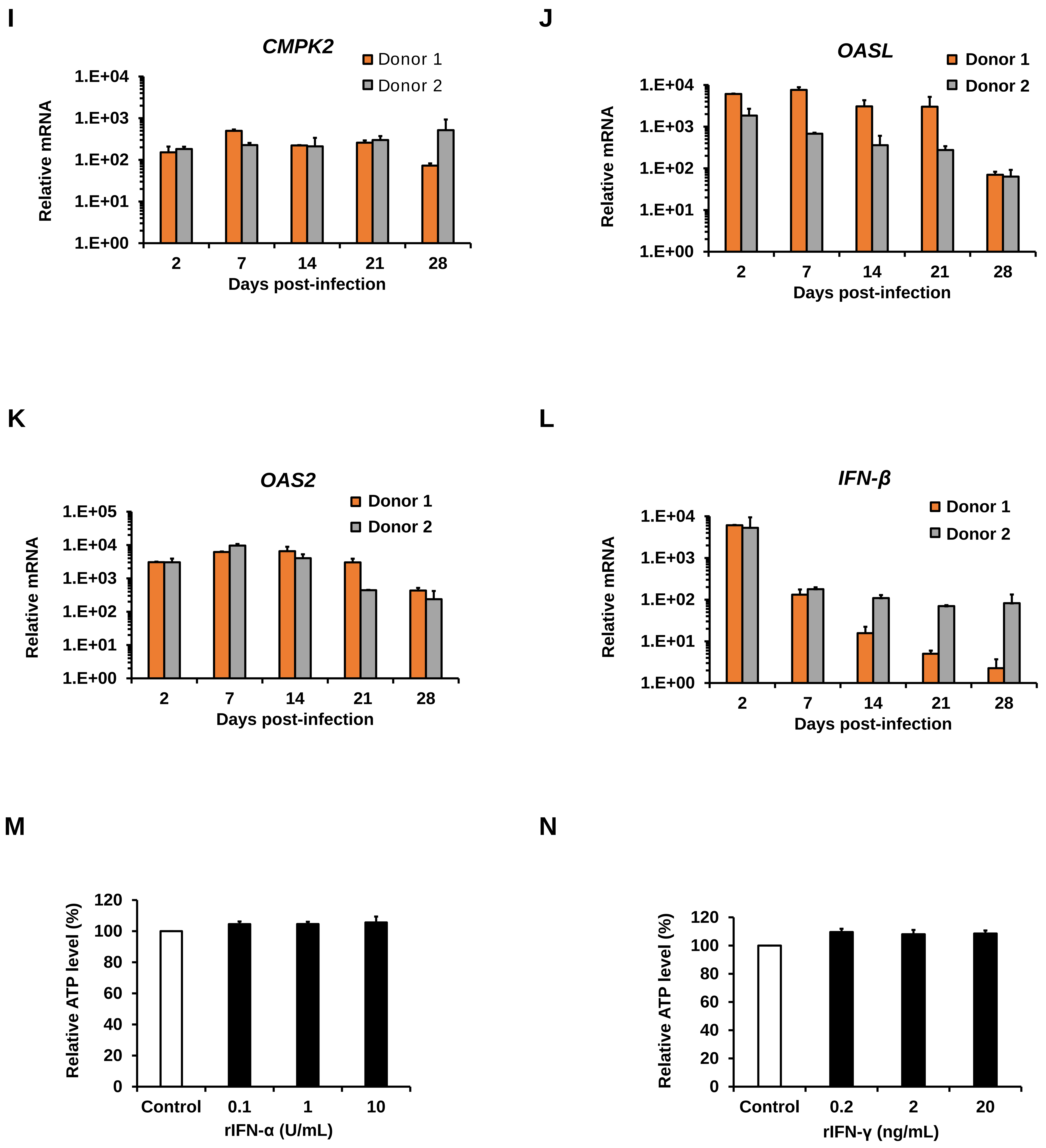
I
CMPK2
Donor 1
Donor 2
Relative mRNA
1.E+04
1.E+03
1.E+02
1.E+01
1.E+00
2
7
14
21
28
Days post-infection
J
OASL
Donor 1
Donor 2
Relative mRNA
1.E+04
1.E+03
1.E+02
1.E+01
1.E+00
2
7
14
21
28
Days post-infection
K
OAS2
Donor 1
Donor 2
Relative mRNA
1.E+05
1.E+04
1.E+03
1.E+02
1.E+01
1.E+00
2
7
14
21
28
Days post-infection
L
IFN-β
Donor 1
Donor 2
Relative mRNA
1.E+04
1.E+03
1.E+02
1.E+01
1.E+00
2
7
14
21
28
Days post-infection
M
Relative ATP level (%)
120
100
80
60
40
20
0
Control
0.1
1
10
rIFN-α (U/mL)
N
Relative ATP level (%)
120
100
80
60
40
20
0
Control
0.2
2
20
rIFN-γ (ng/mL)

Figure S6

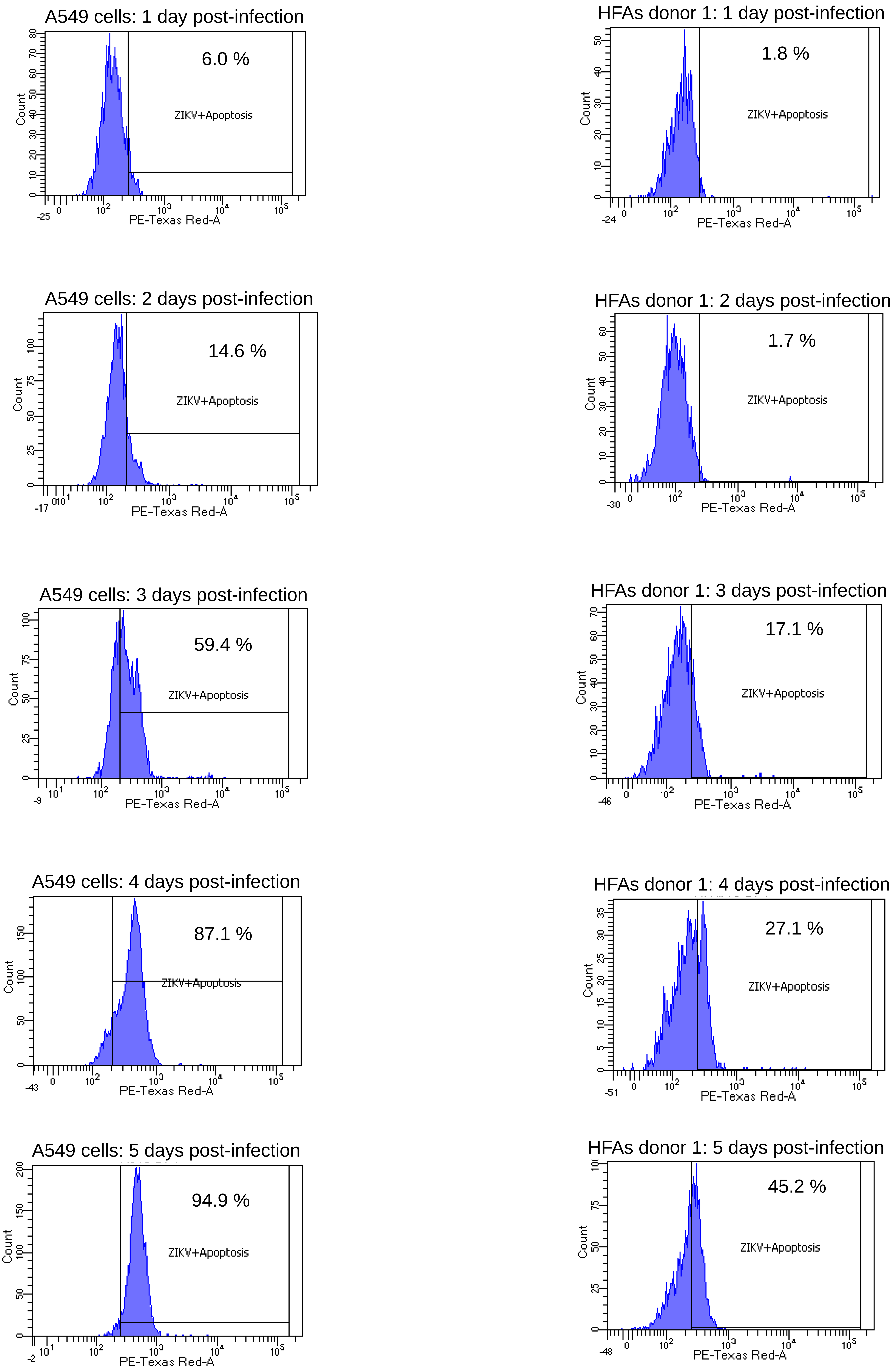
A549 cells: 1 day post-infection
6.0 %
ZIKV+Apoptosis
Count
PE-Texas Red-A
A549 cells: 2 days post-infection
14.6 %
ZIKV+Apoptosis
Count
PE-Texas Red-A
A549 cells: 3 days post-infection
59.4 %
ZIKV+Apoptosis
Count
PE-Texas Red-A
A549 cells: 4 days post-infection
87.1 %
ZIKV+Apoptosis
Count
PE-Texas Red-A
A549 cells: 5 days post-infection
94.9 %
ZIKV+Apoptosis
Count
PE-Texas Red-A
HFAs donor 1: 1 day post-infection
1.8 %
ZIKV+Apoptosis
Count
PE-Texas Red-A
HFAs donor 1: 2 days post-infection
1.7 %
ZIKV+Apoptosis
Count
PE-Texas Red-A
HFAs donor 1: 3 days post-infection
17.1 %
ZIKV+Apoptosis
Count
PE-Texas Red-A
HFAs donor 1: 4 days post-infection
27.1 %
ZIKV+Apoptosis
Count
PE-Texas Red-A
HFAs donor 1: 5 days post-infection
45.2 %
ZIKV+Apoptosis
Count
PE-Texas Red-A

Figure S7

A

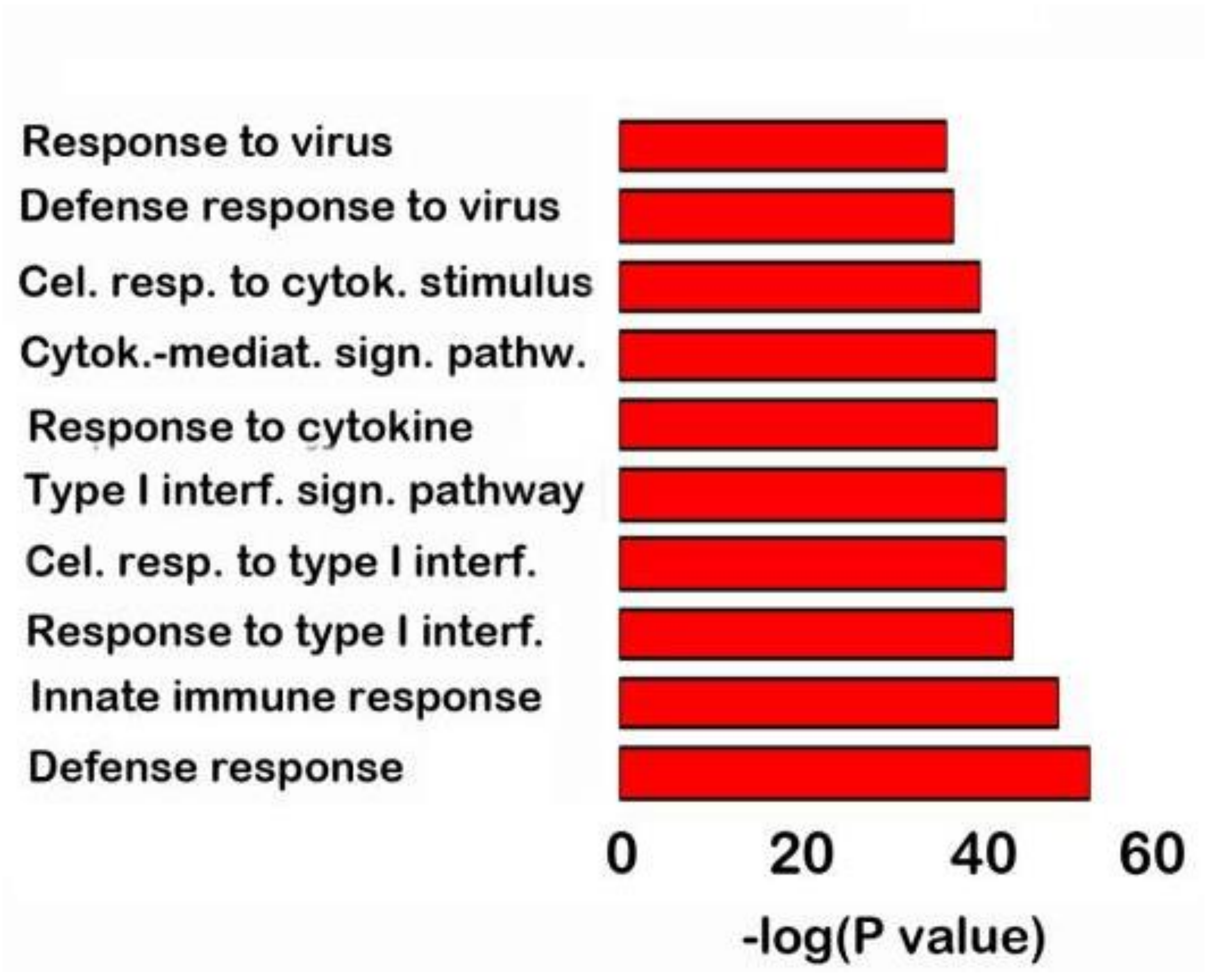
Response to virus
Defense response to virus
Cel. resp. to cytok. stimulus
Cytok.-mediat. sign. pathw.
Response to cytokine
Type I interf. sign. pathway
Cel. resp. to type I interf.
Response to type I interf.
Innate immune response
Defense response
0
20
40
60
-log(P value)

B

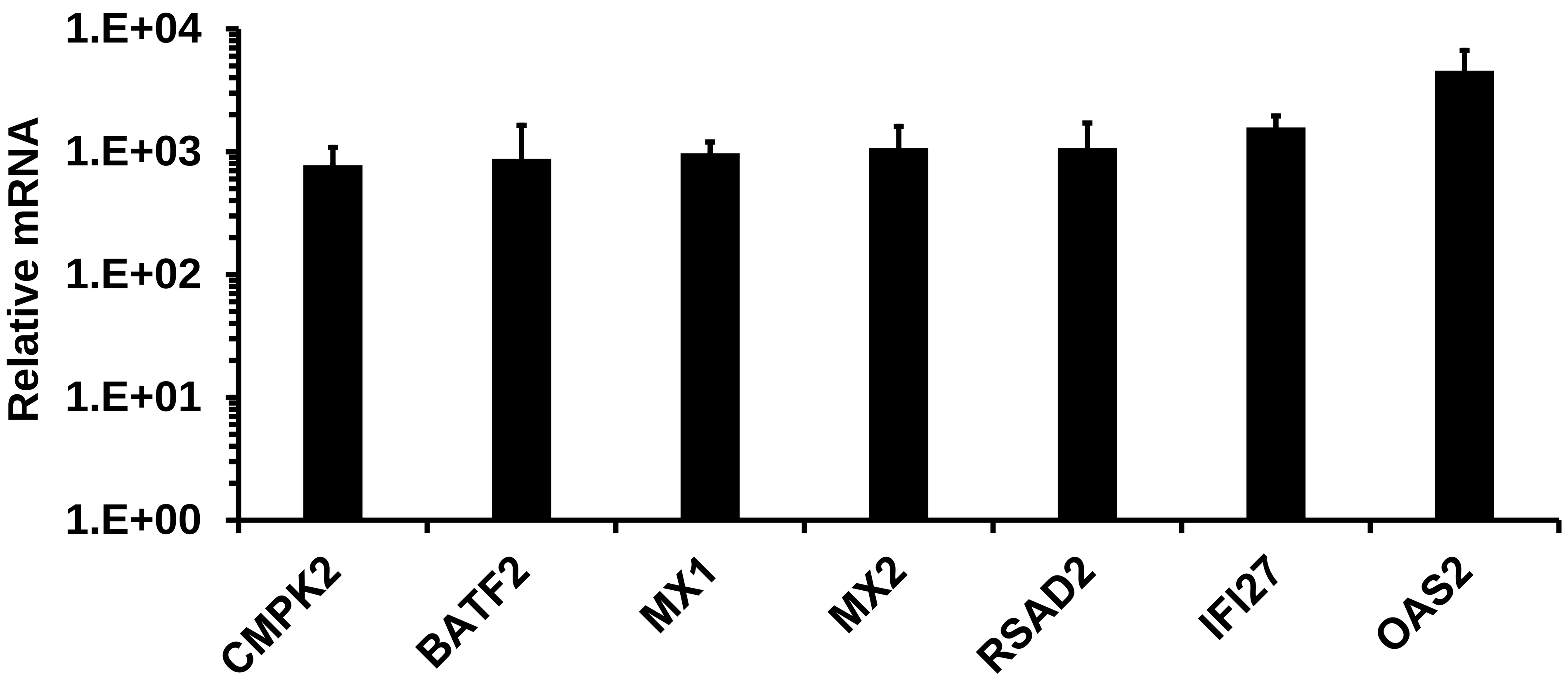
1.E+04
1.E+03
1.E+02
1.E+01
1.E+00
Relative mRNA
CMPK2
BATF2
MX1
MX2
RSAD2
IFI27
OAS2

Figure S8

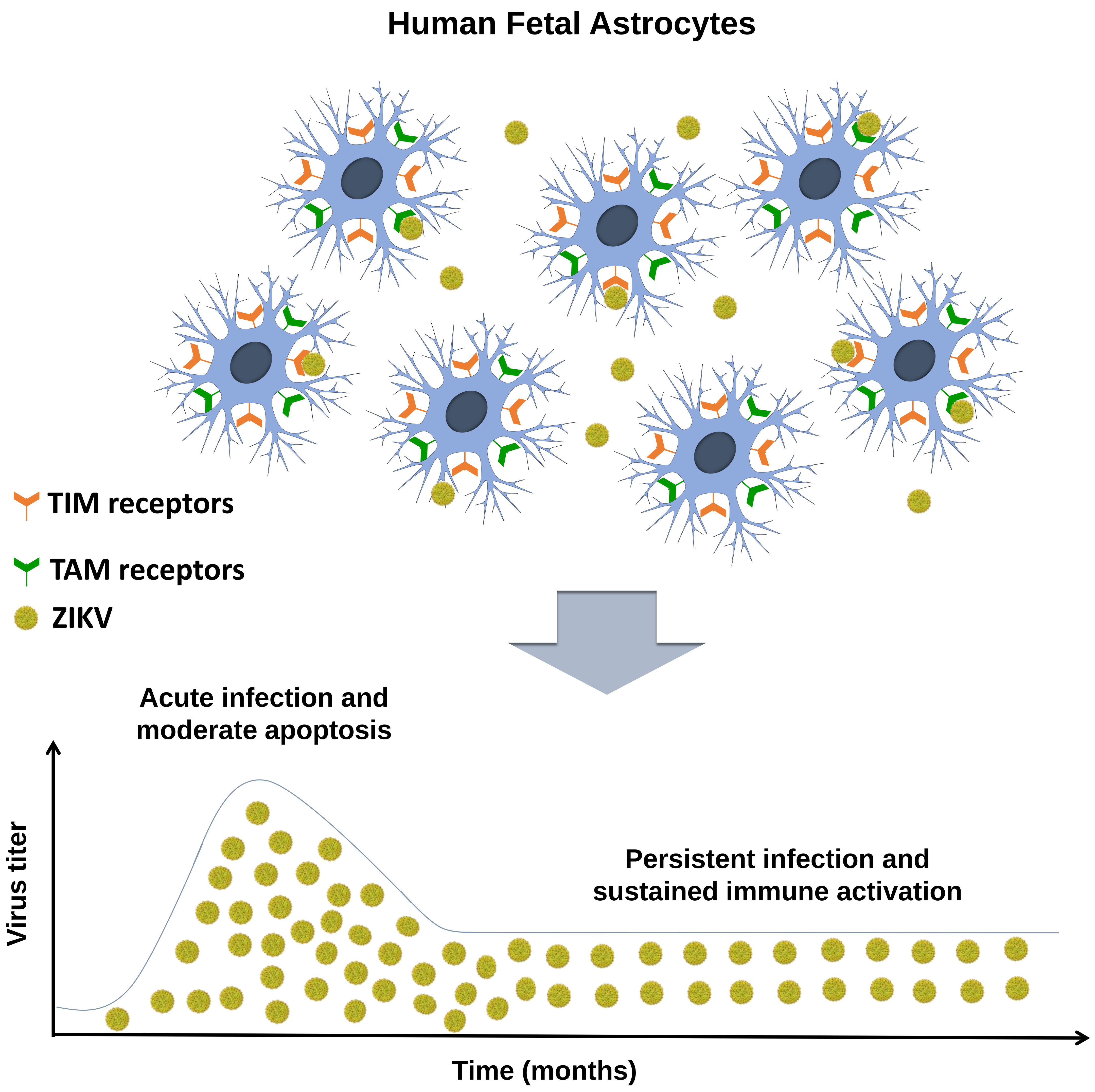

Human Fetal Astrocytes
TIM receptors
TAM receptors
ZIKV
Acute infection and
moderate apoptosis
Virus titer
Persistent infection and
sustained immune activation
Time (months)
